# Isomer-Resolved Mass Spectrometry Imaging Reveals Regional and Age-Dependent Glycolipid Metabolism in Rat Brain

**DOI:** 10.1021/acscentsci.6c00340

**Published:** 2026-07-10

**Authors:** Hsi-Chun Chao, Marisa Asadian, Stanislav S. Rubakhin, Fan Lam, Jonathan V. Sweedler

**Affiliations:** † Beckman Institute for Advanced Science and Technology, 14589 University of Illinois Urbana-Champaign, Urbana, Illinois 61801, United States; ‡ Department of Chemistry, 14589 University of Illinois Urbana-Champaign, Urbana, Illinois 61801, United States; § Department of Bioengineering, 14589 University of Illinois Urbana-Champaign, Urbana, Illinois 61801, United States

## Abstract

Glycolipids are essential for myelin integrity, but their extensive isomeric complexity, where isomers like galactosylceramide (GalCer) and glucosylceramide (GlcCer) have unique functions, has hindered our ability to map their metabolism in the brain. Existing mass spectrometry methods fail to resolve this complexity. We fundamentally overcome this barrier with a powerful new isomer-resolved mass spectrometry imaging workflow. By coupling ion mobility and mass spectrometry imaging with targeted enzymatic pretreatment, our method provides direct visualization of distinct de novo metabolic pathways for GalCer and GlcCer in situ. When applied to rat brains across the lifespan (2, 6, and 12 months), this technology revealed a previously unrecognized age-related decline that selectively affected the GalCer pathway, while the isomeric GlcCer pathway remained comparatively stable. We confirmed these metabolic alterations colocalize with myelin-associated proteins specifically in oligodendrocyte-rich regions, providing a functional link to altered oligodendrocyte-related lipid metabolism. This integrated approach provides a new paradigm for dissecting isomer-specific glycolipid metabolism, offering insight into lipid alterations in brain aging and opening new avenues for biomarker discovery in neurodegeneration.

## Graphical abstract

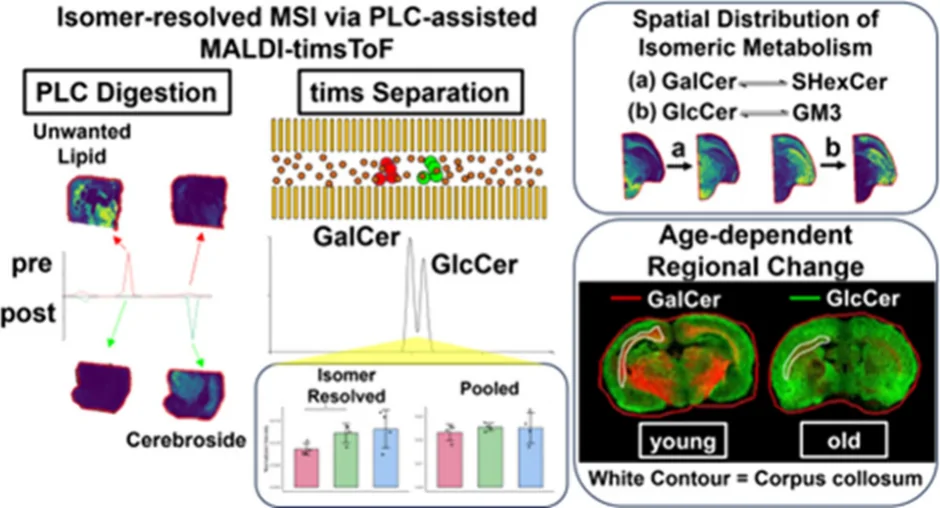

## Introduction

Lipids are among the most chemically complex molecules in the brain, yet existing analytical approaches often fail to resolve the isomerism among lipids, leading to ambiguity in identifying their functions in the brain.^1,2^ Glycolipids, or glycosphingolipids, are one of the most abundant lipid classes in the brain; they are composed of a sphingoid base linked to a glycan headgroup and an esterified fatty acyl chain.^3^ Different glycolipids are classified into different subclasses, including cerebroside (CB or HexCer), lactosylceramde (LacCer or Hex_2_Cer), sulfatides (SHexCer), and ganglioside (sialic acid on the glycan moiety), primarily based on their glycan headgroup.^4^ Specific glycolipids play varied and critical roles in cell signaling and membrane organization associated with neurobiology. In the context of neurodegeneration, aberrant glycolipid metabolism has been implicated in oxidative stress, endosomal–lysosomal dysfunction, endoplasmic reticulum stress, and maladaptive immune responses.^5–7^ While the specific pathogenic mechanisms vary across diseases, converging evidence points to shared metabolic phenotypes, particularly involving CBs, underscoring their central role in neuronal dysfunction.^8,9^ Notably, age-associated shifts in cerebroside metabolism have been linked to altered enzymatic activity between galactosyltransferase and glucosyltransferase, leading to changes in the *de novo* synthesis of isomeric cerebrosides in the brain.^10^ Therefore, elucidating the metabolism and spatial distribution of glycolipids in the brain is essential for understanding their biological functions.

Deciphering glycolipid metabolism in the brain, however, is hindered by their low abundance in complex biological matrices and the structural complexity of their isomeric forms.^11^ Cerebrosides can vary in multiple structural dimensions, including sugar stereochemistry (glucose vs galactose), glycosidic bond configuration (α vs β), and lipid chain composition (Figure 1a).^12^ Overcoming these analytical challenges requires platforms capable of both high sensitivity and in-depth structural resolution. Mass spectrometry (MS) offers the necessary sensitivity and flexibility for glycolipid characterization,^13^ and, when coupled with ion mobility spectrometry (IMS), enables gas-phase separation of isomeric species, providing critical insights into their molecular architecture.^14–17^ Moreover, mass spectrometry imaging (MSI) preserves spatial context while enabling detailed molecular characterization, facilitating region-specific biological investigations (e.g., tissue-level analysis).^18–21^

**Figure 1:**
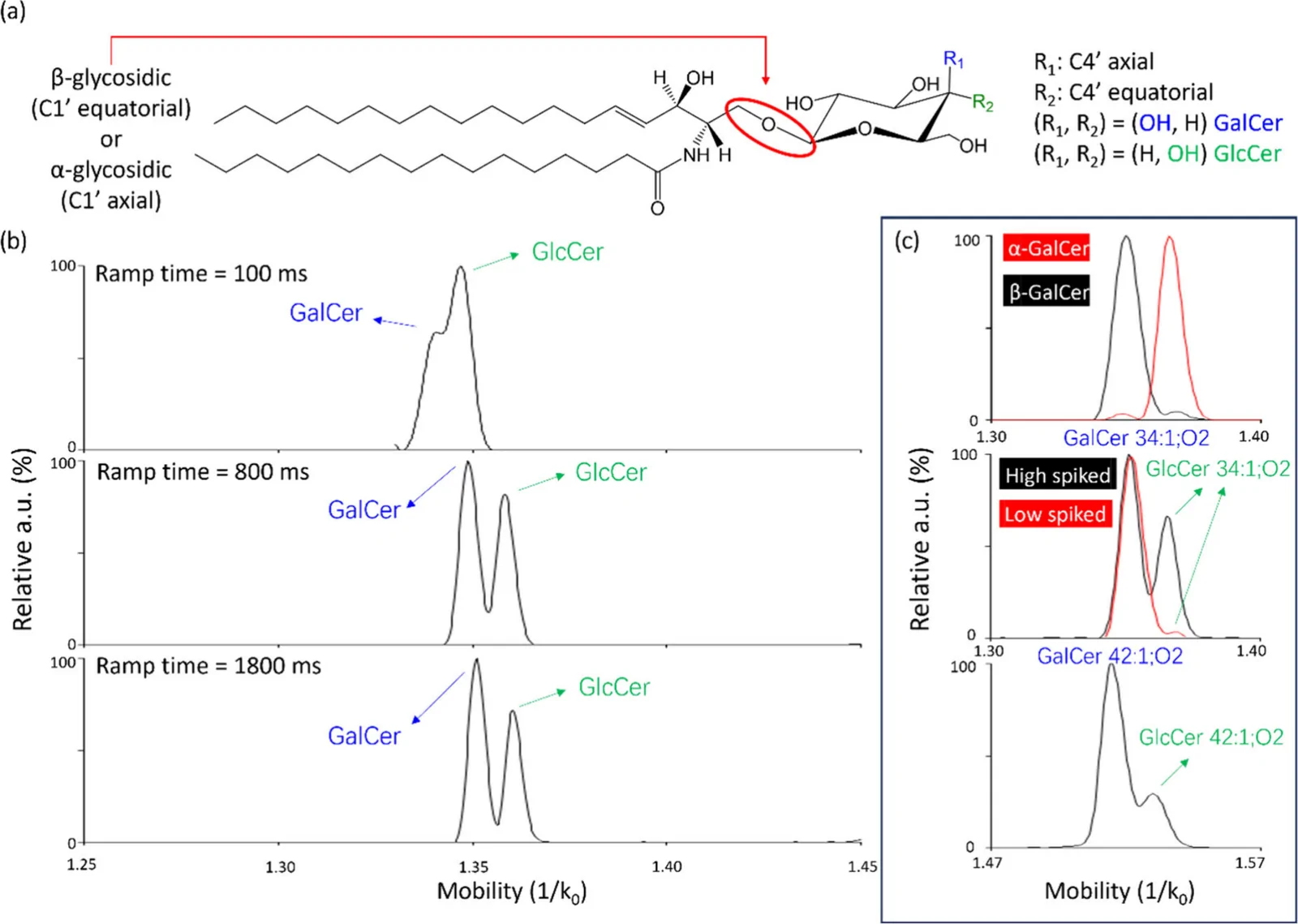
Representative results from TIMS optimization for the differentiation of isomeric cerebrosides. (a) Representative structure of HexCer 34:1;O2, highlighting the β-(1→4)-glycosidic linkage (circled bond). (b) Extracted ion mobilograms (EIMs) of HexCer 34:1;O2 (*m*/*z* 689.558) acquired at varying TIMS ramp times. (c) Representative EIMs illustrating the separation of various isomeric species: α-GalCer 34:1;O2 vs β-GalCer 34:1;O2 (*m*/*z* 689.558); GalCer 34:1;O2 vs GlcCer 34:1;O2 at varying concentrations (∼0.5%); and GalCer 42:1;O2 vs GlcCer 42:1;O2, which differ by the length of the amide-linked fatty acyl chain comparing to HexCer 34:1;O2.

In this work, we established an advanced MSI workflow integrating phospholipase C (PLC) enzymatic digestion and postionization to enhance glycolipid detection, particularly for low-abundance species in brain tissues. PLC selectively cleaves phospholipid headgroups, thereby alleviating ion suppression from abundant glycerophospholipids and improving the ionization efficiency of neutral and acidic glycolipids.^22,23^ Postionization further amplifies lipid signal intensity, including that of minor glycolipid species, by generating additional ions in the postdesorption stage.^24,25^ When coupled with trapped ion mobility spectrometry (TIMS), the workflow achieves separation of key isomeric species, such as glucose- and galactose-based cerebrosides, while preserving high-resolution spatial mapping capabilities in brain sections.^20,26,27^ Leveraging this workflow, we successfully revealed the distinct spatial distributions of isomeric cerebrosides and uncovered their colocalized metabolic pathways within the brain. Applying this workflow to an aging rat brain model, we resolved and mapped the distinct spatial distributions of cerebroside isomers and identified region-specific, age-dependent alterations in their metabolic profiles. These findings not only demonstrate the critical importance of resolving structural isomers for accurate glycolipid characterization but also highlight how such resolution can uncover previously obscured metabolic shifts in the aging brain. Collectively, this work establishes a powerful analytical approach for dissecting isomer-specific lipid metabolism, enabling the investigation of its effects in neurodegeneration and brain aging, and paving the way for deeper mechanistic insights and potential biomarker discovery.

## Results and Discussion

### Establishment of Isomer-Resolved MSI Workflow for Mapping Cerebrosides

Establishing an isomer-resolved MSI workflow requires two major considerations: separation of isomeric species and sufficient sensitivity. Although, IMS has been applied to resolve glycolipid isomers using traveling wave IMS (TWIMS) platforms, including structures for lossless ion manipulations (SLIMs)^14^ and cyclic IMS (cIMS),^16,17^ most biological IMS applications have focused on abundant brain sulfatides or gangliosides,^28–33^ while simpler glycolipid classes such as GlcCer and GalCer have received far less attention. Existing studies often depend on specialized LC–MS workflows^34^ and extensive sample preparation,^35–37^ to resolve cerebroside isomers, making it difficult to move beyond generalized identification under the collective term hexosylceramides (HexCer). Biological applications of MALDI-MSI for cerebrosides are particularly constrained due to difficulties in integrating enrichment protocols with MALDI and in overcoming ion suppression from highly abundant phospholipids.^38^ To overcome these barriers, we established a new PLC-assisted MALDI–TIMS–MSI workflow that integrates enzymatic treatment with high-resolution ion mobility separation to directly image isomeric cerebrosides and their de novo metabolites.

To first assess isomeric differentiation by ion mobility, HexCer 34:1 was examined in both positive and negative ion modes via TIMS. No separation was observed in positive mode, whereas negative mode resolved HexCer 34:1 isomers under default settings, with [HexCer 34:1-H]^−^ showing the strongest signal and selected for further optimization (Figures S2–S3). Systematic optimization of ramp time (50 to1800 ms) and TIMS-in pressure (1.8 mbar to 2.8 mbar) improved mobility resolution 2-fold (FWHM) and achieved a resolving power of ∼150 (at 2.6 mbar) for isomeric HexCer 34:1 (Figure 1b, Figures S4–S5, and Table S2.)

During this optimization, we observed general trends aligned with TIMS resolution theory, where extended ramp times and higher pressure improved separation.^39^ For example, the two ion mobility populations shown in Figure 1b demonstrate that longer ramp times yield improved mobility resolution for the isomeric pair GalCer 34:1;O2 and GlcCer 34:1;O2. However, each parameter required balancing competing factors. While increasing pressure improved mobility resolution, high pressure (2.8 mbar) also led to increased fragmentation, likely from greater ion activation via stronger fields and more frequent collisions. Utilizing 2.8 mbar for TIMS separation also required voltage tuning beyond commercial defaults. Balancing resolution, fragmentation, and operational feasibility, we selected 2.6 mbar. Ramp time, defined as the duration over which the electric field in the TIMS cell is reduced to elute ions according to their mobility, exerted an even stronger influence (Figure 1 and Figure S5), yielding a 2-fold resolution gain at longer durations (>800 ms). This, too, was constrained by fragmentation; GlcCer 34:1;O2 showed increased fragmentation at 1800 ms, consistent with collision energy experiments confirming GlcCer’s more fragile than GalCer (Figure S6). In addition to differential fragmentation, the mobility-dependent intensity shifts may also reflect conformer transitions. We speculate these transitions require collisional stabilization, a process that also showed trends as a function of ramp time, similar to behavior observed in protein ions.^40^ However, further investigation is needed to confirm mechanisms beyond differential fragmentation. Resolution gains plateaued beyond 800 ms, so 800 ms was chosen to maximize separation while maintaining throughput.

Under optimized conditions, TIMS successfully resolved cerebroside isomers based on fine structural differences (Figure 1c). This included separation based on anomeric configuration (α vs β), monosaccharide identity with longer acyl chains (GlcCer vs GalCer in 42:1;O2), and subtle isotopic differences from lipid chain unsaturation (Figure S7). We further performed spiking experiments using porcine brain extracts, where GlcCer 34:1;O2 was not detected. Spike at high and low concentrations further confirmed this robust TIMS selectivity under complex sample conditions.

While TIMS optimization enabled isomeric separation, sensitivity remained a critical bottleneck, as direct tissue analysis yielded little to no detectable signal for many cerebrosides. We tackled this by optimizing tissue preparation with both chemical (e.g., enzyme digestion) and physical (e.g., tissue section thickness) perspective. To begin with, we introduced the first PLC digestion protocol specifically tailored for negative-ion mode glycolipid MSI. This enzymatic treatment cleaves the phosphodiester bond in glycerophospholipids (Figure 2a), removing their polar head groups (phosphate), which represent the primary charged sites for these lipids.^41,42^ By eliminating this charged moiety, abundant glycerophospholipids lose a favorable deprotonation pathway, thereby reducing their ionization efficiency and decreasing competition with other lipid classes, particularly glycolipids, which ultimately alleviates ion suppression. Second, we optimized the physical aspects of tissue preparation, particularly tissue section thickness, as these parameters are critical for spatial fidelity. We used unfixed brain sections to avoid potential lipid loss or cross-contamination,^43^ but found that aqueous digestion, defined here as applying an aqueous enzyme solution directly onto the tissue section, distorted thin (12 μm) brain sections (Figure S8a). Thicker sections (16–20 μm) retained structural integrity; thus, 16 μm was selected as the best compromise between morphological preservation and MALDI-MSI performance, where thinner sections typically aid spatial resolution and mitigate matrix effects.^44^ Washing steps often enhance signal quality; accordingly, a washing step was incorporated after the enzymatic digestion step.^45^ Optimization of washing time (5 and 10 min, Figure 2e and f) and washing buffer concentration (20–100 mM ammonium bicarbonate, Figure S8b) was performed. As a result, a 5 min wash with 50 mM ammonium bicarbonate was adopted, balancing the signal enhancement and tissue fidelity (Prolonged washing time led to tissue distortion during the removal of the bulk wash solution). In addition, the alkaline pH (∼8) of the wash buffer likely enhanced the signal by promoting deprotonation, thus improving desorption/ionization in negative-ion mode.^46^

**Figure 2:**
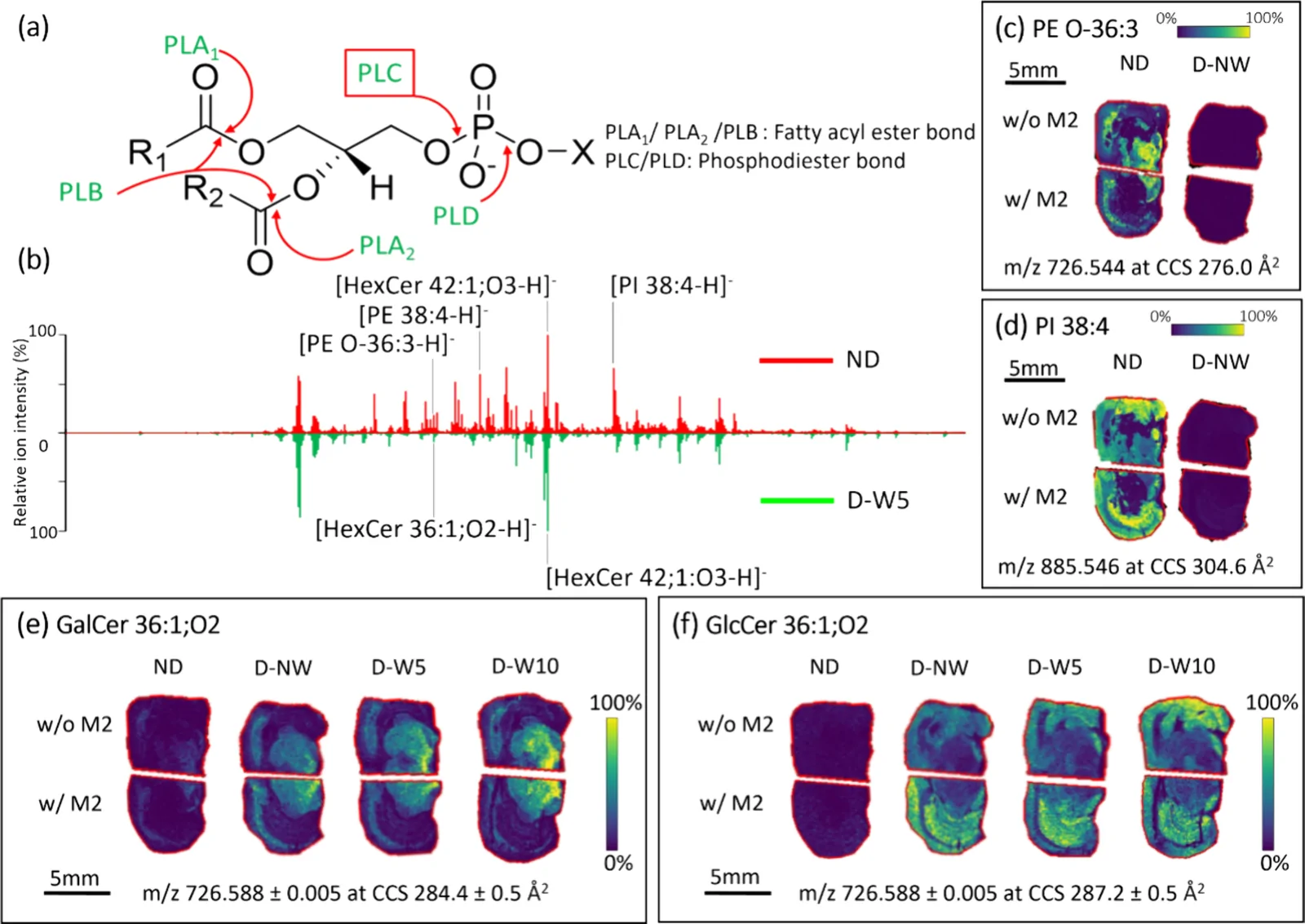
Representative results of PLC enzymatic treatment in MSI analysis of lipid distribution in the brain. (a) Schematic of phospholipase (PL) cleavage sites for different PL subtypes, with the subtype, PLC, used in this study highlighted in red box. (b) Representative average MALDI-MS spectra (*m*/*z* 400–1600) comparing samples without PLC treatment (top, red) and with PLC treatment (inverted bottom, green); zoomed-in *m*/*z* region spectra can be found in Figure S9. All PE and PI species here were annotated solely based on exact mass. (c–f) Extracted ion images generated in SCiLS Lab showing the effects of PLC treatment on (c) PE O-36:3, (d) PI 38:4, (e) GalCer 36:1;O2, and (f) GlcCer 36:1;O2. The color bar represents the relative ion intensities. Abbreviations: PL: phospholipase; PE: phosphatidylethanolamine; PI: phosphainositol; w/: with w/o: without; M2: MALDI-2 laser for postionization; ND: no PLC digestion/treatment; D-NW: with PLC digestion/treatment but without subsequent washing; D-W5 and D-W10: with PLC digestion/treatment followed by 5 or 10 min washing steps, respectively.

This optimized preparation strategy proved highly effective. Representative ion images and mass spectra (Figure 2b–d, Figure S9) confirmed successful digestion, showing the clear removal of abundant glycerophospholipids (e.g., PE and PI) and a simultaneous enhancement of cerebroside signals. These protocol optimizations (Figure 2e–f) enabled the reliable detection of previously obscured minor isomeric species, such as GlcCer 36:1;O2. This success underscores the necessity of carefully balancing tissue morphology, analyte recovery, and ionization efficiency for high-quality MSI.

Beyond tissue preparation, instrument parameters were fine-tuned for maximum glycolipid sensitivity (Table S3). We observed that MALDI-2 postionization only offered a modest sensitivity gain, and this gain was not observed across all species (Figures 2b−f). However, overall signal enhancement differences may have resulted from subtle differences in residual solvent following enzymatic digestion, solution removal, and air drying prior to matrix application, which may have influenced matrix recrystallization behavior and subsequent ionization efficiency. Together, the PLC treatment and MALDI-2 tuning yielded up to an approximately 15-fold signal enhancement based on the ion intensity ratios of the selected species (Table S4). Finally, after optimizing laser field size (Supporting Information discussion), the complete workflow provided a crucial secondary benefit: it not only enhances ionization efficiency by removing abundant phospholipids (e.g., PI 38:4) but also eliminates key isobaric interferences, such as PE O-36:3 (with *m*/*z* close to HexCer 36:1;O2). This removal of interferences is essential for achieving high-fidelity spatial mapping of cerebroside isomers across intact brain sections.

**Figure 3:**
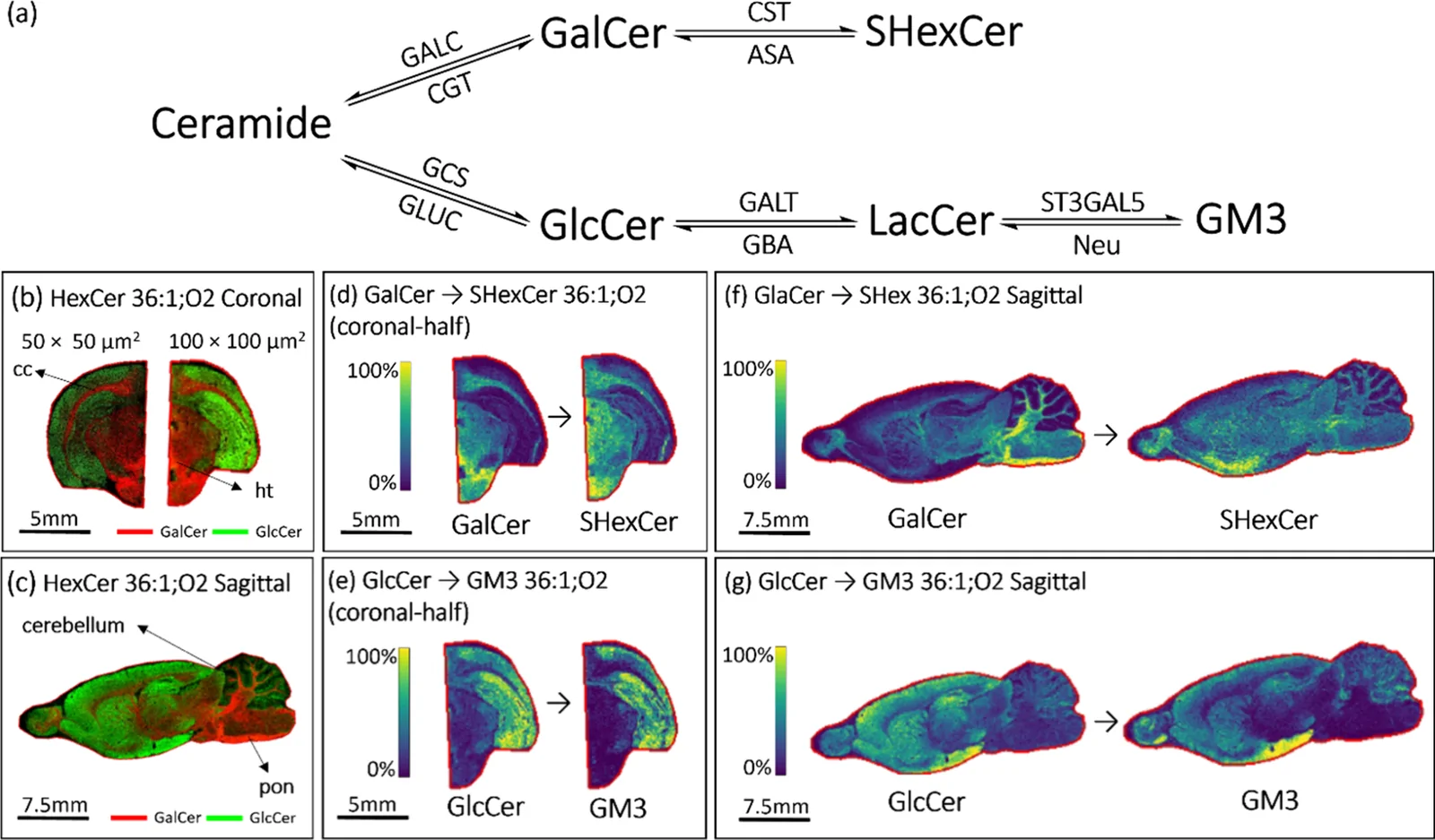
Spatial distribution of isomeric cerebrosides and their corresponding glycolipids in rat brain. (a) General de novo metabolic pathways of glycolipids in mammals. (b–c) Overlaid ion images of isomeric cerebrosides (HexCer 36:1;O2) in (b) coronal and (c) sagittal brain sections, highlighting differential regional distribution; (b) also demonstrates the effect of varying laser sizes on image quality. (d–e) Representative ion images in coronal sections showing isomer-specific metabolism of the same lipid composition (36:1;O2): (d) GalCer → SHexCer and (e) GlcCer → GM3. (f–g) Corresponding images in sagittal sections for (f) GalCer → SHexCer and (g) GlcCer → GM3. Ion images were generated in SCiLS. Abbreviations: GALC, galactosylceramidase; CGT, UDP-galactose-ceramide galactosyltransferase; GCS, UDP-glucose-ceramide glucosyltransferase; GLUC, glucosylceramidase; CST, cerebroside sulfotransferase; ASA, arylsulfatase A; GALT, β-1,4-galactosyltransferase 5/6; GBA, glucocerebrosidase; ST3GAL5, GM3 synthase (gene: ST3GAL5); Neu, sialidase; cc, corpus callosum; ht, hypothalamus.

### Distinct Spatial Distributions of Isomeric Glycolipid Metabolism in Rat Brain

Using our established isomer-resolved MSI workflow (Figure S1), we investigated the spatial organization of glycolipid metabolism in normal rat brains. Although the two de novo metabolic pathways for GalCer and GlcCer originate from the same ceramide precursors (Figure 3a), our workflow provides the first direct visualization of their pronounced spatial separation in situ. Mapping GalCer 36:1;O2 and GlcCer 36:1;O2 across coronal and sagittal sections revealed highly distinct distributions, where differences in field size probed by laser’s beam further affected the ion intensities and image resolution (Figure 3b). GalCer 36:1;O2 was predominantly localized to the corpus callosum (cc) and hypothalamus (ht) in the coronal section, and to the cerebellum and pons in the sagittal section. In contrast, GlcCer 36:1;O2 exhibited a complementary distribution pattern, suggesting functional segregation between the two isomers. Analysis of their respective downstream products, sulfatide (SHexCer 36:1;O2) for GalCer and GM3 36:1;O2 for GlcCer, showed strong colocalization (Figure 3c–f) with only some differences in spatial distribution, indicating that related glycolipid pathways maintain distinct yet overlapping regional patterns. For example, SHexCer 36:1;O2 colocalized with GalCer 36:1;O2 in the coronal section within both the cc and ht. In the sagittal section, SHexCer 36:1;O2 remained enriched in the hypothalamic region but showed comparatively lower signal in the pons and cerebellum relative to its precursor. This observation does not contradict their overall colocalization in the brain but instead suggests differential enzymatic activity within GalCer–SHexCer metabolism. Additionally, Hex2Cer 36:1;O2, which displayed a distribution similar to GlcCer 36:1;O2, was identified as LacCer 36:1;O2, a GlcCer-derived intermediate and precursor to GM3 36:1;O2 (Figure S12). The spatial distributions of highly abundant gangliosides and sulfatides were also consistent with previous literature, further reinforcing our findings regarding the differences in glycolipid metabolism between the two isomeric pathways.^11,47^ Collectively, these findings highlight the region- and pathway-specific organization of glycolipids in the rat brain, underscoring the functional complexity of the isomeric metabolic network. By enabling the simultaneous investigation of multiple metabolites across interconnected isomeric pathways, this workflow demonstrates a strong capability for resolving spatially organized glycolipid metabolism in complex biological systems.

### Profiling of Isomeric Cerebrosides in Aging Rat Brain

Cerebrosides, the simplest glycolipids, exist as two structural isomers (GalCer and GlcCer) that channel into distinct downstream metabolic routes. Leveraging our established PLC-assisted MALDI–TIMS–MSI workflow, we achieved the first direct spatial resolution of GalCer and GlcCer in rat brain tissue, uncovering their distinct regional distributions along with corresponding downstream products (Figure 3). Notably, GalCer showed enrichment in the cc, ht, cerebellum, and pons, regions conventionally reported as highly myelinated, suggesting potential differences in the relationship between glycolipid metabolism and myelination across the isomers. These findings prompted us to investigate how isomeric glycolipid metabolism changes with age, particularly since cerebrosides have been implicated in aging-related processes since the 1950s,^48^ yet significant ambiguity remains regarding their precise roles and regulation across the lifespan. We therefore applied this workflow to profile cerebroside changes in an aging rat brain model (2, 6, and 12 mo). The selected age groups were chosen in accordance with previous reports suggesting relatively slow turnover/long half-lives of glycolipids across species, including humans.^49,50^ This approach allowed us to capture long-term metabolic shifts rather than short-term fluctuations. In total, 37 GalCer and 10 GlcCer species were identified in our samples, corresponding to 47 cerebroside species via MSI (Table S5), of which eight isomeric cerebroside groups were further highlighted in Table S6. Identification criteria are also summarized in the Supporting Information. Figure S10 shows the 2D mobility–*m*/*z* results with the extracted cerebrosides. Five groups were observed with isomeric monosaccharide headgroups, while three other groups likely involved isomerism in the ceramide lipid side chain. As in-depth structural characterization of the ceramide moiety was not performed, cerebroside species were reported based on the total carbon number of the ceramide. However, in mammalian systems, the most commonly observed cerebrosides are expected to comprise a sphingoid backbone containing a d18:1 sphingosine, consistent with the de novo sphingolipid biosynthesis pathway.^3^ We first focused on the global profile and changes across whole brain sections matched to the same brain atlas coordinates among the age groups, comparing the average cerebroside signals across these anatomically consistent sections. Figure 4a shows the heatmap among the rat aging model. Two distinct patterns are observed: one in which cerebrosides are generally elevated in 2 mo rats and decrease with age, and another in which certain cerebrosides increase in 12 mo rats, an age-dependent upward trend. Figure 4b presents the PCA results for the three age groups, revealing distinct separation and a clear age-related trend. The first two principal components account for a large portion of the variance (PC1 = 64.9%, PC2 = 15.6%), and the corresponding loading scores are provided in Table S7. To assess the significance of individual cerebrosides across age groups, all ion signals were first normalized to the total ion signal, followed by Welch’s ANOVA, with FDR-corrected *p*-values reported in Table S8. A total of 19 features showed significant differences (Welch’s ANOVA, *p* < 0.05; FDR = 10%), comprising 16 GalCer and 3 GlcCer species. To further examine pairwise group differences, both the Games-Howell test and FDR-adjusted (Benjamini-Hochberg) *t* tests were conducted, with results summarized in Table S8. Among the GalCer species, 15 and 6 showed significant differences (*p* < 0.05) between 2 mo and 6 mo in the Games-Howell and Benjamini-Hochberg tests, respectively, while 16 and 5 GalCer were significant between 2 mo and 12 mo. In contrast, the three GlcCer species were only significant in the Games-Howell test between 2 mo and 6 mo and did not show significance in other pairwise comparisons or statistical tests. A few isomeric species were found to be significant (*p* < 0.05), including GalCer 36:1;O2, GlcCer 36:1;O2, and GalCer 38:1;O2, although GlcCer 38:1;O2 did not show any significant differences among the three age groups (Figure 4c).

**Figure 4:**
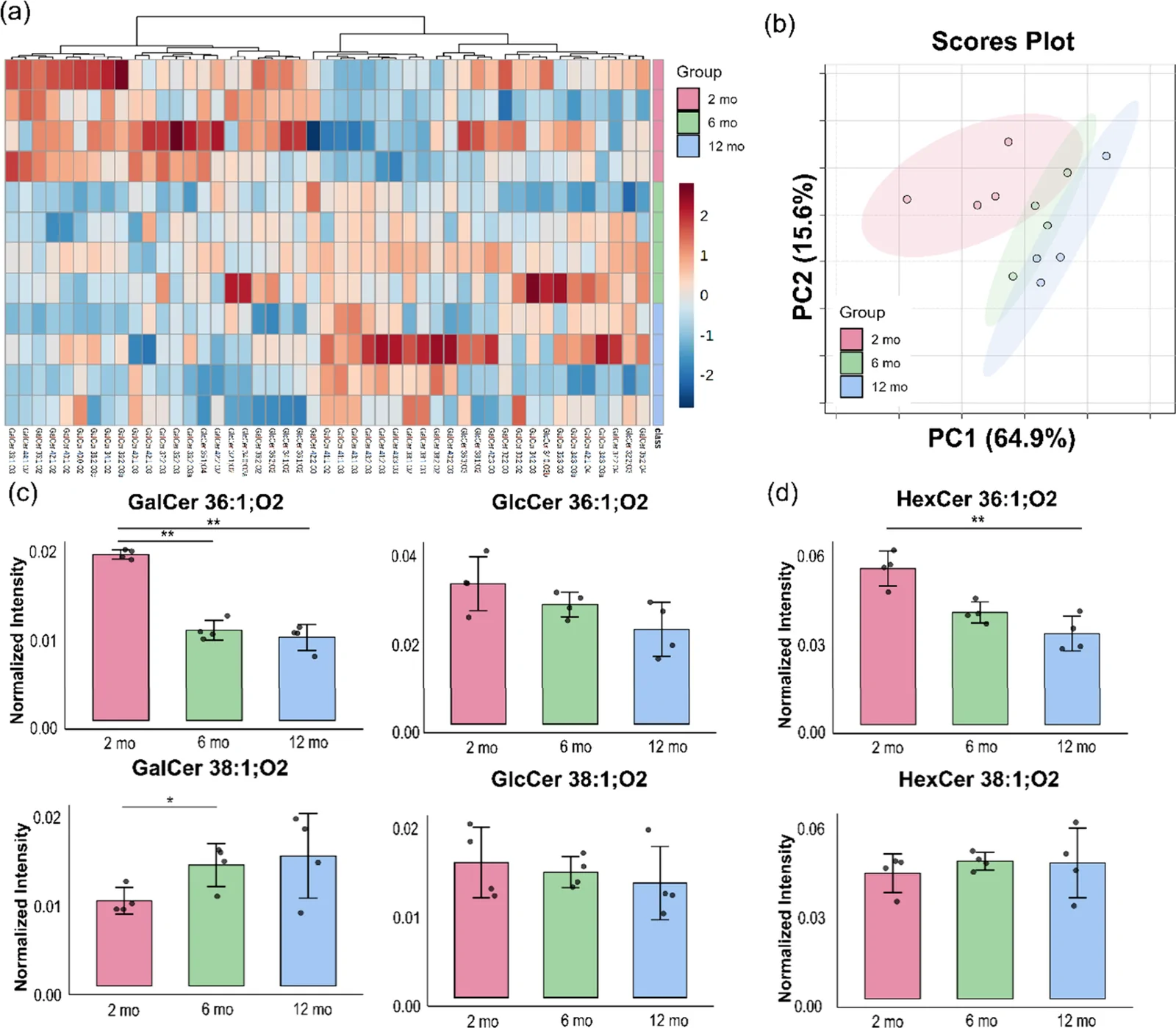
Age-related global changes in profiled isomeric cerebrosides in rat brain (whole coronal sections). (a) Heat maps showing total cerebroside profiles from all samples (*n* = 4 per group; 2, 6, and 12 mo). Cerebrosides are clustered using Ward’s method. (b) PCA plot (with 95% confidence interval withing groups) distinguishing the three age groups. (c) Bar plots for representative isomeric pairs, GalCer 36:1;O2 vs GlcCer 36:1;O2, and GalCer 38:1;O2 vs GlcCer 38:1;O2, across ages. (d) Bar plots of pooled results for HexCer 36:1;O2 and HexCer 38:1;O2. Each species was normalized to the total ion counts (TIC). Error bars are representing standard deviations. **p < 0.01 and *p < 0.05 in both Games-Howell tests and FDR-adjusted (Benjamini–Hochberg) *t* tests.

To evaluate the impact of isomer resolution, we pooled the corresponding isomers and reapplied the same statistical analyses (Welch’s ANOVA, Games-Howell test, and FDR-adjusted *t* tests), with results summarized in Table S9. As shown in Figure 4d, pooling the isomeric features led to a loss of significance between 2 mo and 6 mo for both HexCer 36:1;O2 and HexCer 38:1;O3 after FDR adjustment. These findings highlight the importance of resolving and distinguishing isomeric species during data acquisition and analysis.

To confirm these profiling results, a subsequent set of analyses (*n* = 3) was performed on adjacent brain sections (mostly consecutive, with a maximum separation of 0.5 mm) from the same animals. This sampling strategy allowed us to corroborate our observations while minimizing biological variation and maintaining strict anatomical consistency within the same brain region. Across these samples, a total of 45 cerebrosides were detected. The lipids closely matched that observed in the initial set, supporting the reproducibility and robustness of the reported findings. The only exception was one isomeric group, HexCer 34:1;O2, which fell below the identification threshold in the validation set. Importantly, pairwise comparisons using the Games–Howell test (Figure S11) showed that the significant differences for GalCer 36:1;O2 and GlcCer 36:1;O2 were consistently reproduced in the validation set, whereas GalCer 38:1;O2 displayed a similar trend but did not reach statistical significance (*p* = 0.2). Together, these validation data reinforce the technical robustness and reproducibility of the workflow, while further supporting that the observed age-related cerebroside differences reflect consistent biological features rather than section-specific variation.

Beyond the isomeric species, notable patterns emerged in relation to lipid side chain length. GlcCer was detected only in long-chain cerebrosides (total carbon < 40). In contrast, GalCer generally exhibited age-related decreases among long-chain species (except 38:1, Figure 4b), but showed an increasing trend in odd-numbered very-long-chain cerebrosides (total carbon ≥ 40).

To this end, we found significant changes in isomeric cerebroside global profiles among the three age groups, 2 mo, 6 mo, and 12 mo (Figure 4a). As previously reported, GalCer species dominated, while GlcCer remained minor components in mammalian brains.^51^ Figure 4b revealed clear separation between the groups via PCA, following a trend consistent with age (red to green to blue), suggesting that cerebroside composition undergoes gradual, age-dependent alteration. Notably, changes between 6 mo and 12 mo were less pronounced, indicating that cerebroside composition may stabilize after early adulthood. Chain length-specific modulation emerged as a prominent feature. Sphingolipids with defined fatty acyl chain lengths are linked to distinct neuronal pathways,^52^ largely due to the substrate specificity of ceramide synthases (CerS1–S6).^53,54^ Interestingly, we observed opposing trends between long-chain and very-long-chain GalCer species across ages, suggesting differential ceramide synthase activity during development and maturation (Figure 4a). Similar inverse patterns in fatty acyl chain profiles have been associated with both neurodegenerative disease^55^ and ages,^56^ where long-chain ceramides promote pro-apoptotic signaling, whereas very-long-chain species support neuroprotection.^57^ Our results generally align with previous reports in aged brain on global cerebroside profiles, which are traditionally obtained via whole-brain homogenization. This demonstrates that our platform allows for robust cerebroside profiling with the added capability of spatial isomeric differentiation. Furthermore, these results reinforce the concept that chain length diversity within cerebrosides reflects functional specialization in the brain.

### Isomeric Glycolipid Metabolisms and Regional Changes in Aging Rat Brains

We next focused on the metabolisms between the isomeric cerebroside pair, GalCer 36:1;O2 and GlcCer 36:1;O2, due to the significant changes observed in the global cerebroside profile as a function of age. GalCer 36:1;O2 decreased significantly with age (*n* = 4, *p* < 0.001 in Welch’s ANOVA and Games–Howell; *p* < 0.05 in Benjamini–Hochberg), while GlcCer 36:1;O2 also declined, albeit more modestly (*n* = 4, *p* < 0.05 in both Welch’s ANOVA and Games-Howell tests). Overlaid ion images of GalCer 36:1;O2 and GlcCer 36:1;O2 from higher-resolution coronal section analyses across all three age groups (Figure 5a) revealed age-dependent changes in the relative ion intensities of isomeric cerebrosides, showing spatial patterns consistent with those described in Figure 3. To extend these findings, we profiled additional sphingolipid species within the de novo biosynthetic pathway beyond cerebrosides (Figure 3a). These included SHexCer 36:1;O2 derived from GalCer, as well as LacCer 36:1;O2 and ganglioside GM3 36:1;O2 derived from GlcCer. Whole-brain quantitative analysis (*n* = 4, pairwise Games–Howell *t* tests) showed that SHexCer 36:1;O2 decreased significantly between 2 mo and 6 mo (*p* < 0.01), while LacCer 36:1;O2 exhibited a more gradual decline, reaching significance only between 2 mo and 12 mo (*p* < 0.05) (Figure S12 and Table S10). The spatial distributions of the larger glycolipids were consistent with previous literature,^11,47^ and the trends paralleled those observed for the isomeric cerebrosides.

**Figure 5:**
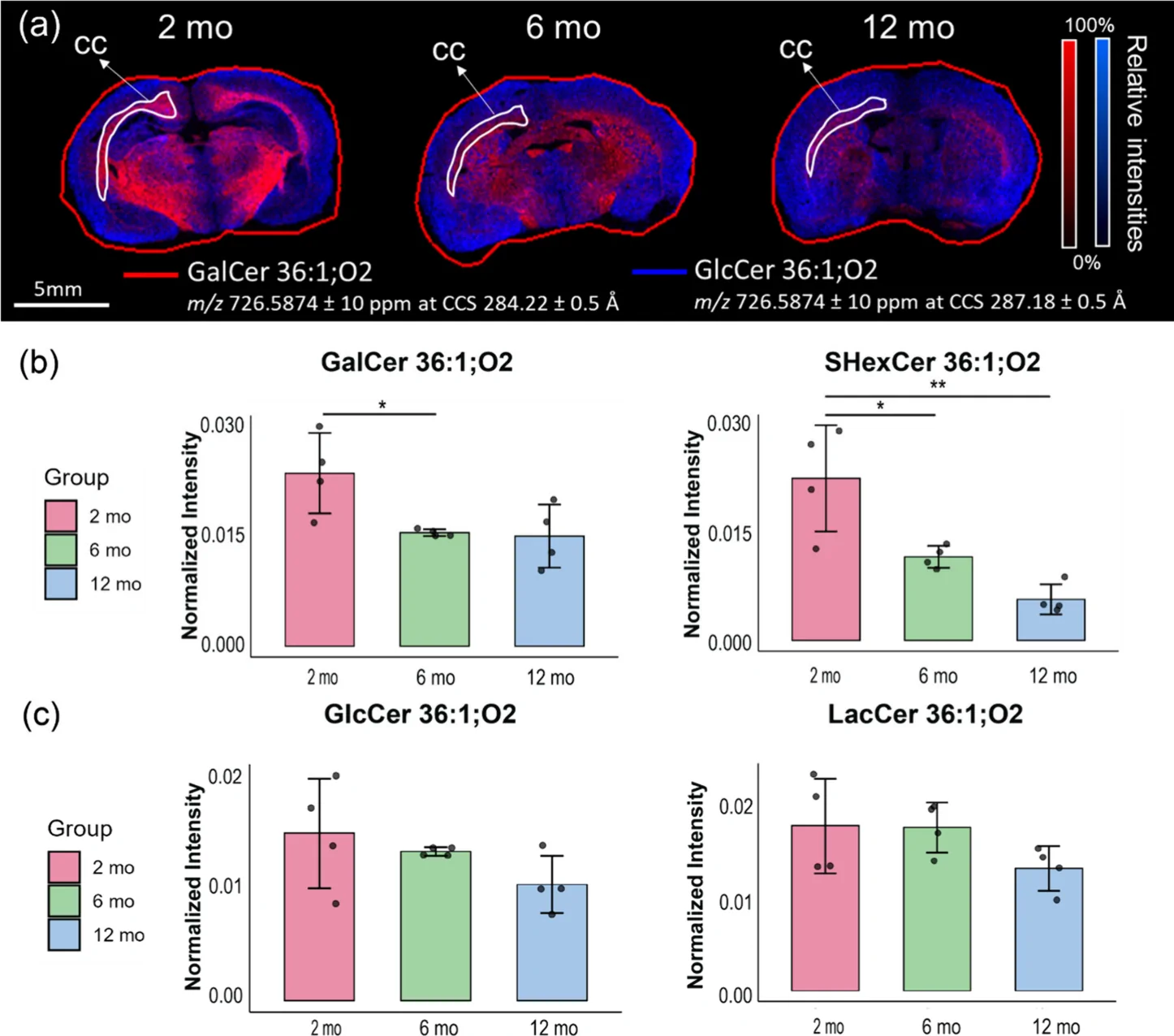
Isomeric glycolipid metabolism and regional distribution in aging rat brains. (a) High-spatial-resolution overlaid ion images of isomeric GalCer 36:1;O2 (red) and GlcCer 36:1;O2 (green) in coronal brain sections from 2 mo, 6 mo, and 12 mo old rats. Overlaid signals were normalized to each individual ion but not across isomers; therefore, the images reflect relative spatial distributions within each age group rather than direct intensity comparisons between isomers. Bar charts comparing (b) GalCer 36:1;O2 and SHexCer 36:1;O2 and (c) GlcCer 36:1;O2 and LacCer 36:1;O2 within the corpus callosum (cc) region, highlighted by the white contour in panel (a) (representative regions). Each species was normalized to the total ion counts (TIC). Error bars represent standard deviations. **p < 0.01 and * *p* < 0.05 by Games–Howell test *t* test.

Given the strong spatial component of cerebroside distributions, we next performed region-specific analyses. In the corpus callosum (cc, also see optical images in Figure S13), GalCer 36:1;O2 levels were markedly reduced with age (*p* < 0.01, Table S11), accounting for much of the decrease observed in whole-brain profiles (Figure 5b). (Note that while statistical comparisons were performed using strictly matched brain atlas coordinates, the high-resolution images in Figure 5a depict a slightly different anatomical plane due to tissue exhaustion; see Figure S13 for details.) SHexCer 36:1;O2 also showed significant age-related declines in the cc, suggesting a region-specific vulnerability in this pathway. In contrast, GlcCer 36:1;O2 and its downstream products (LacCer and GM3, Figure 5c and Figure S14) displayed no significant differences among age groups within this region. This regional divergence underscores the necessity of isomer-resolved analysis to capture localized glycolipid metabolism, revealing distinct vulnerabilities that would be obscured in aggregate profiles.

To contextualize these findings within broader lipid biology, age-related declines in cerebrosides have been reported and are consistent with our observation of lower levels in aged brains.^58^ However, the functional implications of cerebroside isomerism in aging and neurodegenerative disease remain underexplored. Compared to gangliosides and sulfatides, cerebrosides have historically received less attention, partly due to their lower abundance relative to downstream glycolipids (with the exception of GalCer 42:1;O3, a dominant cerebroside species in mammalian brain).^59^ Recent advances in spatial lipidomics have begun to reveal region-specific changes in gangliosides and sulfatides, emphasizing the need for a more comprehensive “glycolipidomics” approach.^11,60^ Yet, profiling isomeric cerebrosides has remained a critical gap, underscoring the importance of analytical platforms capable of resolving these molecules. Our workflow addresses this gap by unraveling the isomerism among these fundamental glycolipids and capturing their associated metabolic transformations, providing a comprehensive view of glycolipid metabolism within the brain.

Building upon these spatial observations, we next explored the potential functional implications of isomeric cerebroside alterations. To this end, we performed mass-tag immunolabeling of myelin-associated proteins. The results demonstrated strong colocalization of GalCer species with oligodendrocyte-rich, myelinated regions (Figure S15), particularly emphasizing the isomeric cerebrosides GalCer 36:1;O2 and SHexCer 36:1;O2, which colocalize with myelin-associated proteins in aged rat brains and exhibit significant, region-specific reductions. Such decreases likely reflect region-specific impairments in oligodendrocyte function, which may result from localized dysfunctional myelination. The mechanistic relevance of these changes is supported by studies in Cgt knockout mice, where loss of the CGT enzyme, which is required for GalCer biosynthesis, still permits myelin formation but leads to abnormal electrophysiological properties, including increased radial impedance, indicative of compromised nerve conduction.^61^ Although isomer-specific analysis was not explicitly performed, cerebroside disruption has also been implicated in multiple system atrophy, a Parkinsonian disorder, where reductions in both SHexCer and tentatively annotated GalCer destabilize myelin and may alter α-synuclein handling in oligodendrocytes.^62^ Complementary evidence from conditional sulfatide knockout models further supports that glycolipid perturbations can compromise both myelin sheath stability and axonal integrity, as sulfatide depletion in adult-onset mice impairs axonal function and induces degeneration independent of overt myelin loss.^63^ Collectively, these findings point to a critical role for precise glycolipid metabolism, particularly in maintaining functional myelin.

Delving deeper into the temporal dimension of these changes, we also observed a sharp decline in SHexCer 36:1;O2 between 2 mo and 6 mo, corresponding to early adulthood in rats, suggesting vulnerability of glycolipid homeostasis during a period traditionally considered neurochemically stable. Wu et al. showed that glycolipid transfer protein (GLTP) is highly enriched in the growing myelin sheath during early brain development, where oligodendrocyte-specific *Gltp* knockouts lead to ER abnormalities, hypomyelination, and reduced myelin glycolipid content, underscoring GLTP’s role in ER-to-myelin lipid trafficking during active myelin assembly.^64^ Therefore, disruption of this transfer pathway during early life myelination could set the stage for region-specific vulnerabilities later manifest as structural and metabolic deficits in aging brains.

While GalCer- and SHexCer-associated changes dominated our observations, less pronounced alterations were detected in GlcCer and its downstream glycolipids. Nevertheless, pathological GlcCer accumulation is a hallmark of Gaucher’s disease due to GBA loss of function,^65^ with documented regional variation in GBA expression in the brain.^66^ Oligodendrocyte-specific GBA1 inactivation disrupts myelination, induces neurodegeneration, and affects lipid homeostasis, highlighting that GlcCer buildup in oligodendrocytes can have severe functional consequences.^67^ This isomer-resolved MSI workflow could detect such accumulations and dissect isomer-specific glycolipid metabolism across diverse physiological and pathological contexts. Taken together, these findings illustrate that myelin-associated glycolipid composition is not static but dynamically regulated across the lifespan, with vulnerabilities emerging well before overt neurodegeneration.

A minor limitation of our workflow is the potential for matrix effects to occur during the MSI process.^18^ To address this, we utilized total ion count (TIC) normalization, the most commonly adopted strategy to account for inter- and intrabatch pixel-by-pixel signal variations.^68^ As demonstrated across our quantitative analyses (e.g., bar graphs), this approach enabled reliable comparisons across different samples. Furthermore, strategies to mitigate batch effects are detailed in the Methods and Supporting Information. While we acknowledge that residual matrix effects may still be present, a major advantage of our workflow is the incorporation of on-tissue enzymatic digestion using PLC. This step significantly reduces ionization suppression for targeted analytes, and thereby minimizes matrix effects, by removing highly abundant glycerophospholipids from the tissue sections, further allowing the detection and visualization of minor isomeric cerebroside components (Figure 2). Additionally, orthogonal validation using parallel analytical techniques (e.g., LC-MS) was not performed, largely due to the requirement for specialized instrumentation and methods to achieve isomeric differentiation, as noted in the Introduction. Our imaging approach provides the distinct advantage of simultaneously resolving both the spatial and isomeric complexity of biosynthetic glycolipids, bridging a critical gap between molecular specificity and anatomical context. This level of resolution not only advances our understanding of lipid metabolism in development, aging, and disease, but also provides a blueprint for identifying early, region-specific glycolipid changes that could serve as biomarkers or therapeutic targets.

## Conclusions

This work establishes a new MSI workflow integrating PLC treatment, postionization, and TIMS separation, as a robust approach for high-sensitivity, isomer-resolved imaging of glycolipids directly from brain tissues. By achieving mobility separation of cerebroside isomers differing in sugar moiety, we resolved the spatial and metabolic organization of GalCer- and GlcCer-based pathways. Applied to an aging rat model, we revealed distinct regional and age-dependent shifts in glycolipid metabolism, most notably in GalCer 36:1;O2 and its downstream sulfatide, implicating specific oligodendrocyte-related pathways in early and region-specific reductions. The integration of isomer-resolved MSI with spatial immunolabeling of myelin-based protein linked these metabolic patterns to anatomical context, underscoring the value of combining molecular and morphological information. By directly mapping isomer-specific glycolipid networks in situ, this workflow opens a path toward disentangling the biochemical underpinnings of brain aging and neurodegeneration and provides a framework for identifying localized metabolic alterations in glycolipids as potential biomarkers.

## Methods

### Tissue Preparation with PLC Digestion

Coronal and sagittal rat brain sections (16 μm thick) were cut and mounted onto ITO-coated glass slides (Delta Technologies, Loveland, CO). All brains were sectioned after minimal storage time and mounted onto glass slides, then stored at –20 °C prior to further preparation. Enzymatic treatment and matrix application were freshly performed before MSI acquisition. For enzymatic treatment, ∼50 μL of a 50 U mL^–1^ PLC solution was evenly aliquoted onto each tissue surface and incubated at 37 °C for 30 min. After incubation, the solution was removed via vacuum suction, and the slides were air-dried. A gentle wash with 50 μL of 50 mM ammonium bicarbonate was performed for 5 min. Isomeric standard mixture, GalCer 36:1;O2 and GlcCer 36:1;O2, at 50 μg mL^–1^ were spotted onto each slide for reference. DAN matrix (20 mg mL^–1^ in 70:30 (v/v) ACN/H_2_O) was applied using the HTX M5 Sprayer (HTX Technologies, Chapel Hill, NC) under the following parameters: 4 passes, 100 μL min^–1^ flow rate, 60 °C nozzle temperature, 60 mm spray distance, ∼10 psi pressure, and 1500 mm min^–1^ spray velocity. Slides were stored briefly in a nitrogen chamber before MSI acquisition. Detailed chemical, animal, and sample preparation protocol can be found in the Supporting Information.

### Isomer-Resolved MSI

MSI data were acquired using a MALDI-2 timsTOF fleX mass spectrometer (Bruker Corp., Billerica, MA), equipped with a SmartBeam 3D laser system operating at 355 nm for MALDI. Second 266 nm laser was used for MALDI-2 postionization. The system was controlled using timsControl and flexImaging software. Detailed instrument parameters, including voltage settings and TIMS conditions, are provided in Table S3. For imaging sagittal sections and data sets used for statistical analysis (*n* = 3 or 4 per age group; total of 21 sections), an M5 Small or M5 Small M2 laser beam settings with Beam Scan was used at a 100 × 100 μm^2^ field size to minimize acquisition time. Higher-resolution imaging of coronal sections was performed using the same beam at a 50 × 50 μm^2^ field size. Spectra were acquired at 1 kHz with 50 laser shots per pixel, laser power set at 70%, and a postionization delay of 5 μs. To reduce intrabatch variation and potential artifact, brain sections from different age groups were imaged in alternating order (Table S12). Prior to each acquisition, external mass calibration was performed using an ESI tuning mix with enhanced quadratic regression (Agilent Technologies, Santa Clara, CA). Mobility calibration was conducted in ESI mode using linear regression, with detection maintained within 0.5% variation across a 0.01 Da window. Standard spots were also ablated before and after each batch to monitor *m*/*z* and trapping stability. In addition, adjacent QC regions from another brain section were ablated between whole-tissue imaging runs to ensure signal consistency. Mass-tag MSI and its related sample preparation protocol, and data analysis can be found in the Supporting Information.

## Supplementary Material



## Data Availability

Raw image data will be available via request. All study data are included in the article and/or Supporting Information.

## Notes

This includes the PLC-assisted MSI workflow (Figure S1); additional extracted ion mobilograms from optimization (Figures S2, S4–S5); additional experimental results from the optimization of tissue preparation, including ion images and mass spectra (Figures S3, S6–S9); a 2D mobility-*m*/*z* plot for profiling isomeric cerebrosides (Figure S10); additional ion images and boxplots for glycolipid metabolism in aging rat brains (Figures S11–S12, and S14); overlaid optical images with brain atlas (Figure S13); mass-tag ion imaging results (Figure S15); enlarged heatmap (Figure S16); abbreviation table (Table S1); tables for ion mobility resolution, detailed mass spectrometry parameters, signal enhancement, and profiled isomeric cerebrosides, statistical results (Tables S2–S12); and additional materials, methods, and discussion (Supporting Text) (PDF)
