# Supplementary material for: Isomer-Resolved Mass Spectrometry Imaging Reveals Regional and Age-Dependent Glycolipid Metabolism in Rat Brain
Source: ACS Cent Sci. 2026 Jul 10;12(8):1146–57. doi: 10.1021/acscentsci.6c00340 (PMC13523495; doi:10.1021/acscentsci.6c00340)
Supplement: Supplementary file 1 [file oc6c00340_si_001.pdf]

Supporting Information for

Isomer-Resolved Mass Spectrometry Imaging Reveals Regional and Age-Dependent Glycolipid Metabolism in Rat Brain

Hsi-Chun Chao[1,2], Marisa Asadian[1,2], Stanislav S. Rubakhin[1,2], Fan Lam[1,3], and Jonathan V. Sweedler[1,2,*]

[1]Beckman Institute for Advanced Science and Technology, University of Illinois Urbana-Champaign, Urbana, IL 61801, USA
[2]Department of Chemistry, University of Illinois Urbana-Champaign, Urbana, IL 61801, USA
[3]Department of Bioengineering, University of Illinois Urbana-Champaign, Urbana, IL 61801, USA

*Address correspondence to Dr. Jonathan V. Sweedler
Department of Chemistry
University of Illinois Urbana−Champaign
600 S. Mathews Ave.
Urbana, IL 61801, USA
Email: jsweedle@illinois.edu

Table of Contents

Supporting Figures

Fig. S1. Schematic of the isomer-resolved MSI workflow for mapping isomeric glycolipid metabolisms in rat brains....3
Fig. S2. Extracted ion mobility distributions of isomeric GlcCer and GalCer 34:1;O2 obtained using default IMS settings under positive and negative ion modes. 4
Fig. S3. Initial results for matrix and targeted ion-type selection.. 5
Fig. S4. Extracted ion mobility distributions of HexCer 34:1;O2 (m/z 689.558) acquired at varying TIMS-in pressures....6
Fig. S5. Extracted ion mobility distributions of HexCer 34:1;O2 (m/z 689.558) acquired at ramp time.. 7
Fig. S6. MS2 spectra of isomeric cerebrosides....8
Fig. S7. Extracted ion mobility distributions of HexCer 42:1;O2 (m/z 810.682) and HexCer 42:2;O2 (m/z 808.660) acquired under the optimized TIMS condition 9
Fig. S8. Additional results from the opimization of sample preparation and washing.. ...10
Fig. S9. Representative zoomed-in m/z spectra before and after PLC treatment with a washing step 11
Fig. S10. 2D ion mobility–m/z spectrum with extracted cerebroside (CB) species. 12
Fig. S11. Bar plots of representative isomeric pairs, GalCer 36:1;O2 vs. GlcCer 36:1;O3 and GalCer 38:1;O2 vs. GlcCer 38:1;O2, across ages (2 mo, 6 mo, and 12 mo) from biological replicates (n = 3). 13
Fig. S12. Downstream glycolipids from the de novo metabolic pathway to isomeric cerebrosides....14
Fig. S13. Overlaid optical images and corresponding rat brain atlas sections....15
Fig. S14. Comparison of GM3 36:1;O2 in the cc region. 16
Fig. S15. Ion images of HexCer 36:1;O2 and a mass-tag target for myelin-associated proteins. 17

Supporting Tables

Table S1. Abbreviations 19
Table S2. Calculated FWHM resolving power values for GlcCer and GalCer. 20
Table S3. Detailed optimized instrumental settings. 21
Table S4. Signal enhancement factors derived from Figure 2. 22
Table S5. Profiled isomeric cerebrosides in aged rat brains 23
Table S6. Highlighted isomeric cerebrosides in aged rat brains. 25
Table S7. Loading scores of the first two principal components from PCA. 26
Table S8. The statistic results of isomeric cerebrosides among three aged groups, 2 months (2 mo), 6 months (6 mo), and 12 months (12 mo) (n=4)....29
Table S9. The statistic results from pooled cerebrosides. 32
Table S10. The statistic results of the downstream glycolipids. (n=4). 34
Table S11. The statistic results from the regional analysis in the cc (n = 4). 34
Table S12. Imaging acquisition order used to minimize artificial batch effects. 34

Supporting Texts

Supporting Texts....35
with SI references

Supplementary Figures

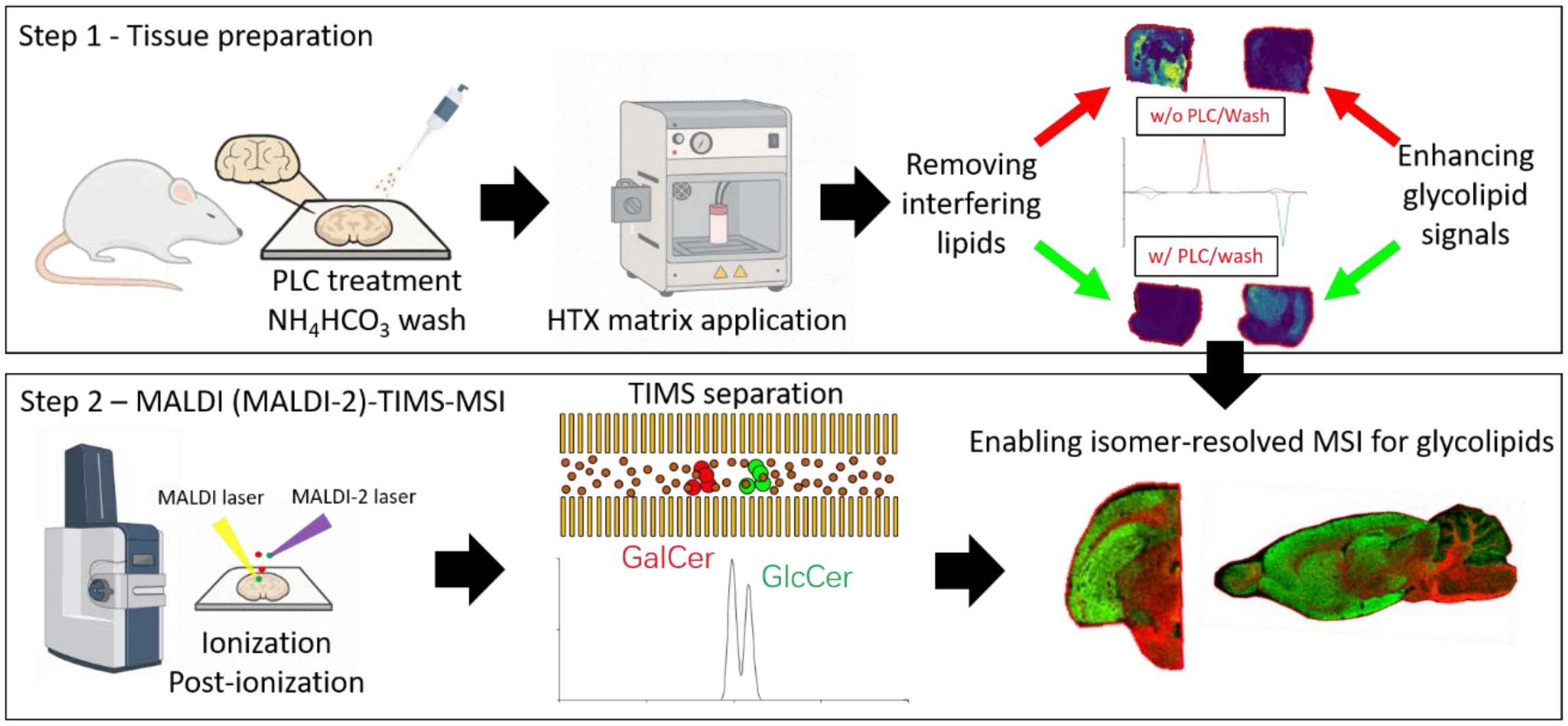

Fig. S1. Schematic of the isomer-resolved MSI workflow for mapping isomeric glycolipid metabolisms in rat brains.

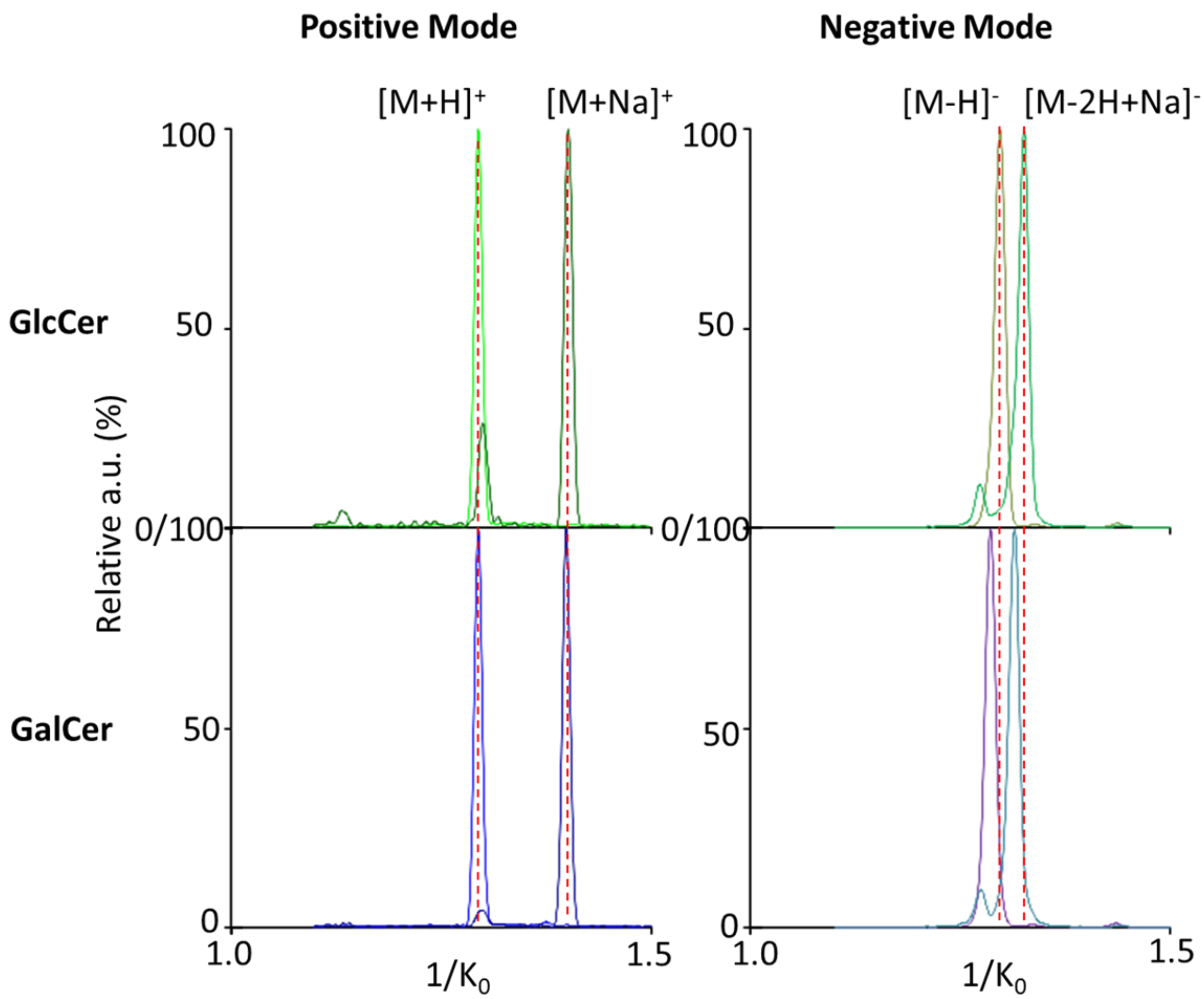

Fig. S2. Extracted ion mobility distributions of isomeric GlcCer and GalCer 34:1;O2 obtained using default IMS settings under positive and negative ion modes. Standards were analyzed at a concentration of 50 μg mL^{-1}. In positive mode, the common MALDI-generated species included protonated and sodiated ions, while in negative mode deprotonated and doubly deprotonated sodium adducts were observed. Red dashed lines indicate the mobilities of GlcCer 34:1;O2 across these ion types, which were then compared to the corresponding ion types of GalCer 34:1;O2. The resulting ion mobility distributions highlight distinct mobility differences between the two isomeric species.

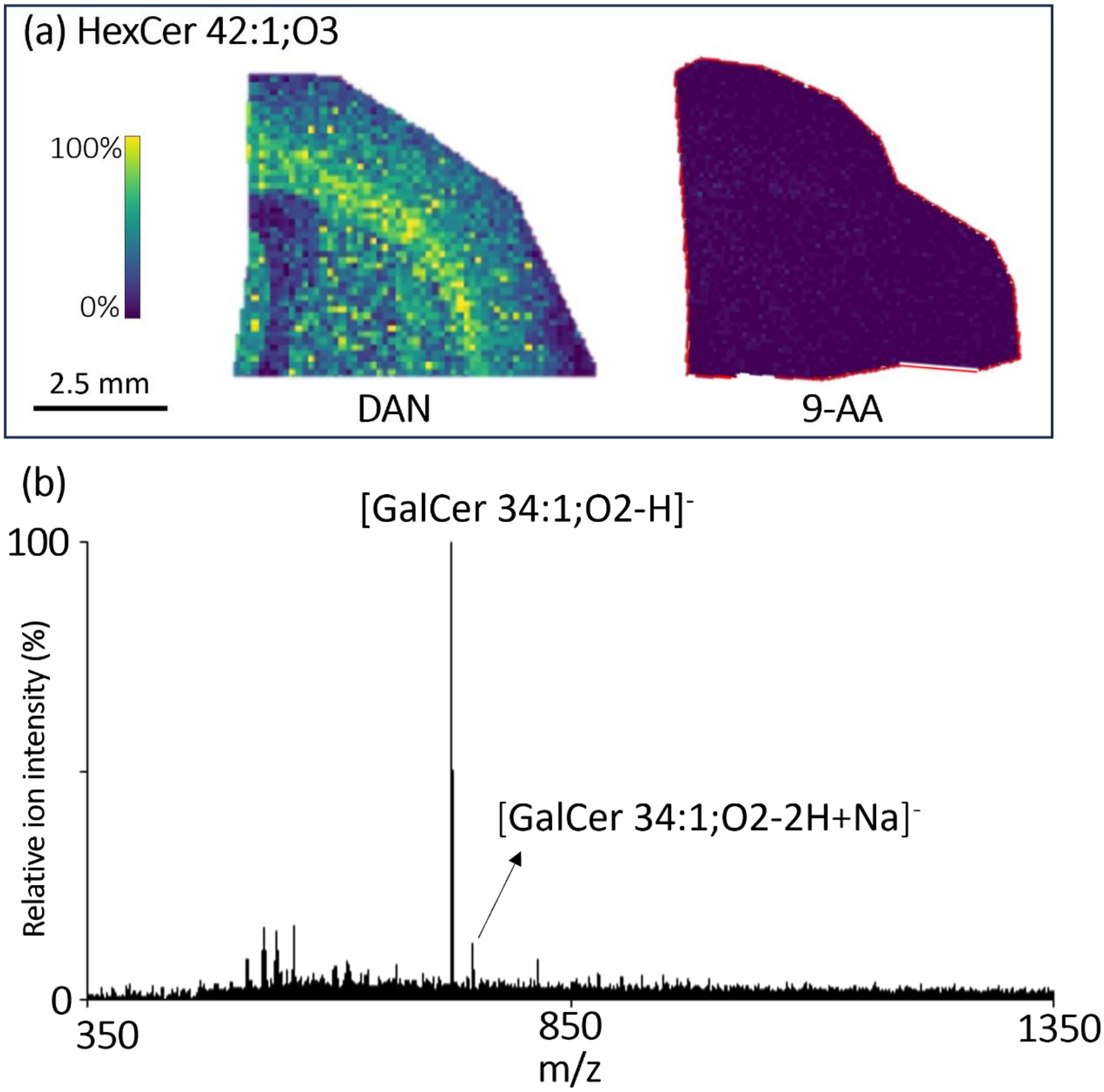

Fig. S3. Initial results for matrix and targeted ion-type selection. (a) Comparison of ion intensities in rat brain tissue using 1,5-diaminonaphthalene (DAN) and 9-aminoacridine (9-AA) matrices, shown for the high-abundance cerebroside HexCer 42:1;O_3 (m/z 826.68). In contrast, the endogenous isomeric species HexCer 34:1;O_2 could not be observed in either matrix under these initial conditions. (b) Representative mass spectrum acquired using 1,5-diaminonaphthalene (DAN) as the MALDI matrix with a 50 μg mL^{-1} GalCer 34:1;O2 standard. The spectrum reflects direct analysis of the standard itself, not spiked onto a pre-coated matrix surface, and was used as the reference for method optimization.

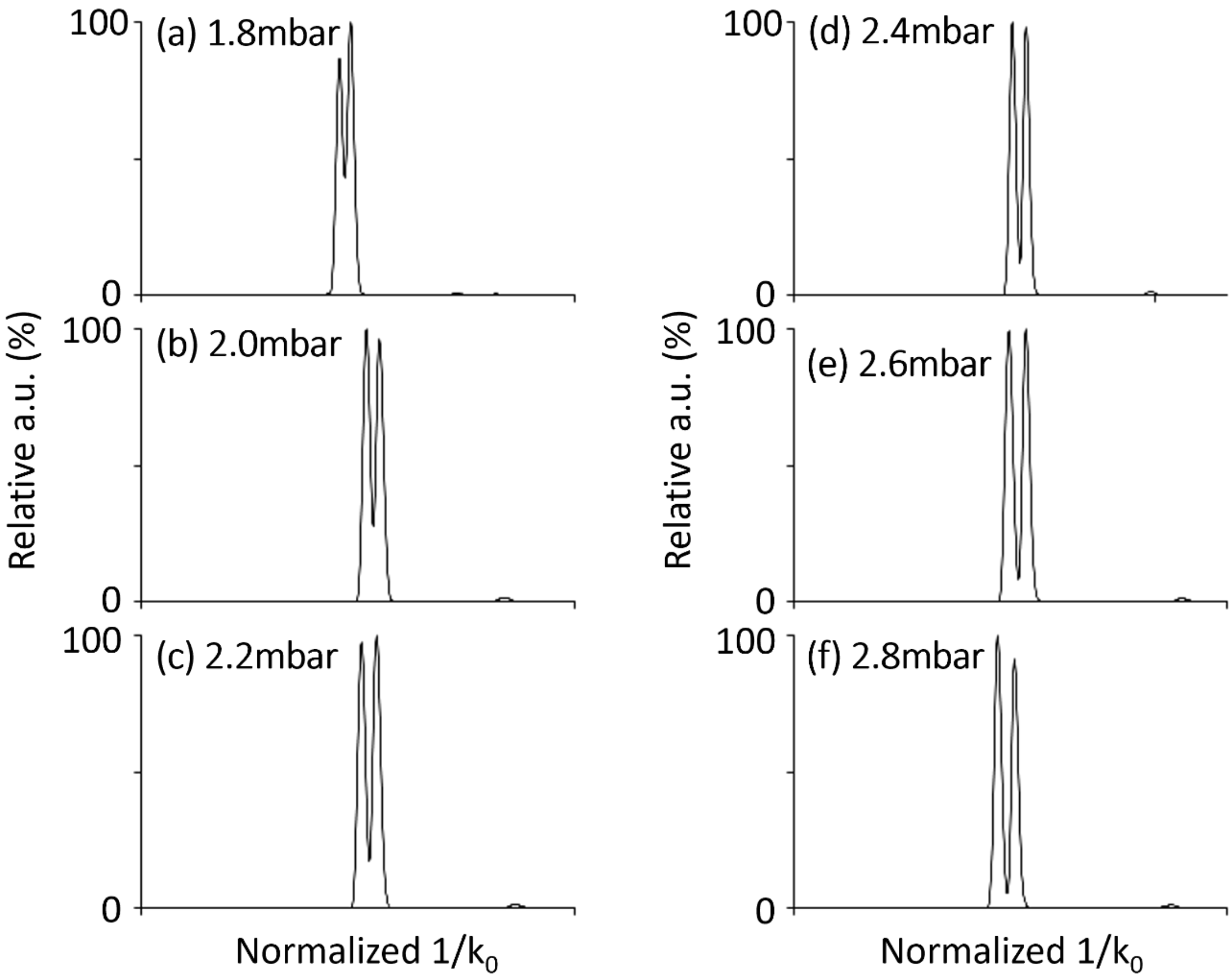

Fig. S4. Extracted ion mobility distributions of HexCer 34:1;O2 (m/z 689.558) acquired at varying TIMS-in pressures. For visualization, the $1/k_0$ values under each condition were normalized to center the two peaks. A ramp time of 600 ms was used. Calculated full width at half maximum (FWHM) and resolving power (Rp) values for GlcCer and GalCer were listed in Table S1.

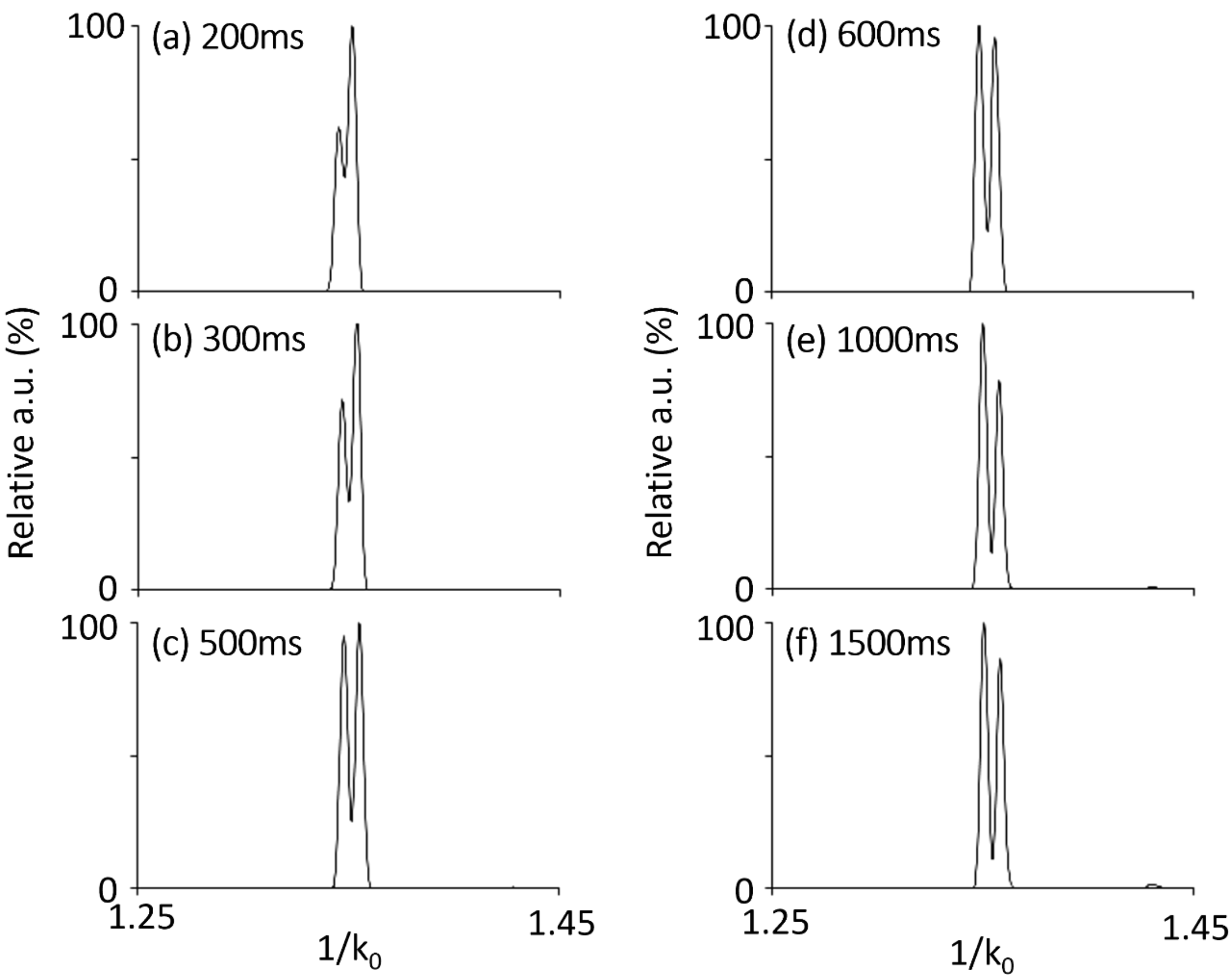

Fig. S5. Extracted ion mobility distributions of HexCer 34:1;O2 (m/z 689.558) acquired at different ramp time. The Calculated full width at half maximum (FWHM) and resolving power (Rp) values for GlcCer and GalCer were listed in Table S1. 2.6 mbar was used as pressure.

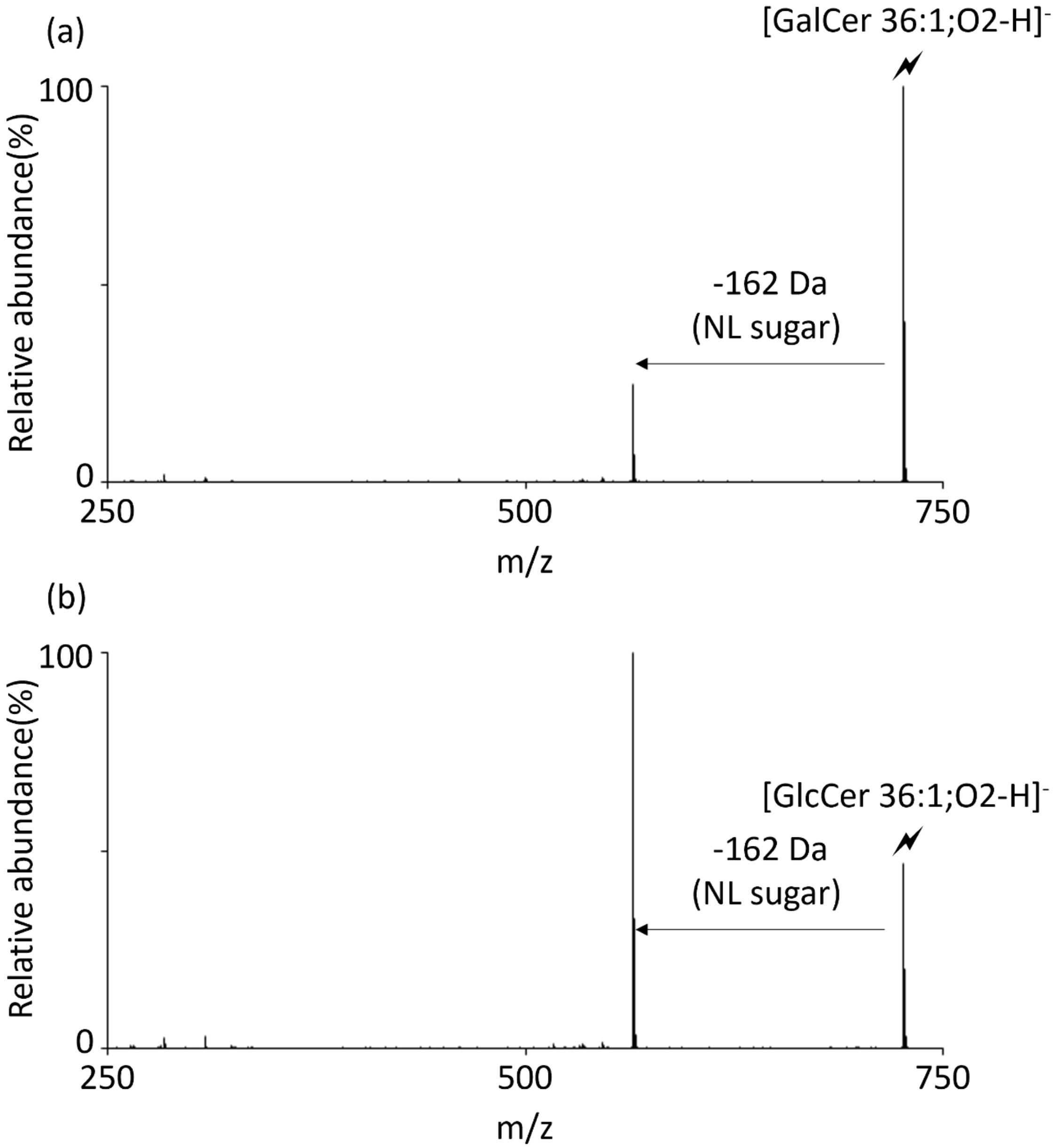

Fig. S6. MS2 spectra of (a) GalCer 36:1;O2 and (b) GlcCer 36:1;O2 under identical collisional energy (CE=35 eV).

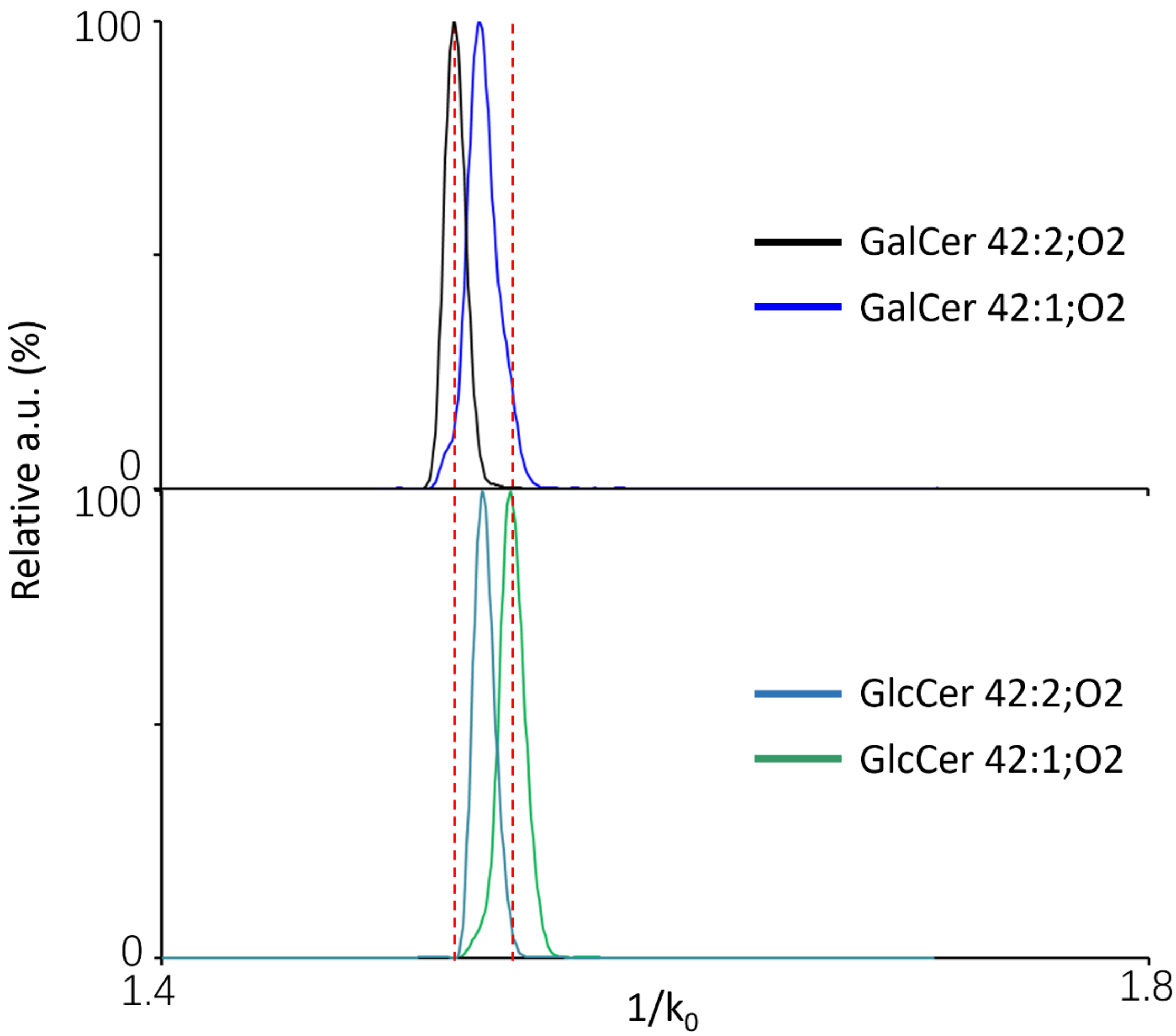

Fig. S7. Extracted ion mobility distributions of HexCer 42:1;O2 (m/z 810.682) and HexCer 42:2;O2 (m/z 808.660) acquired under the optimized TIMS condition, demonstrating separation according to the degree of unsaturation in cerebroside lipid compositions. Red dashed lines indicate the mobility differences between the two isomeric species.

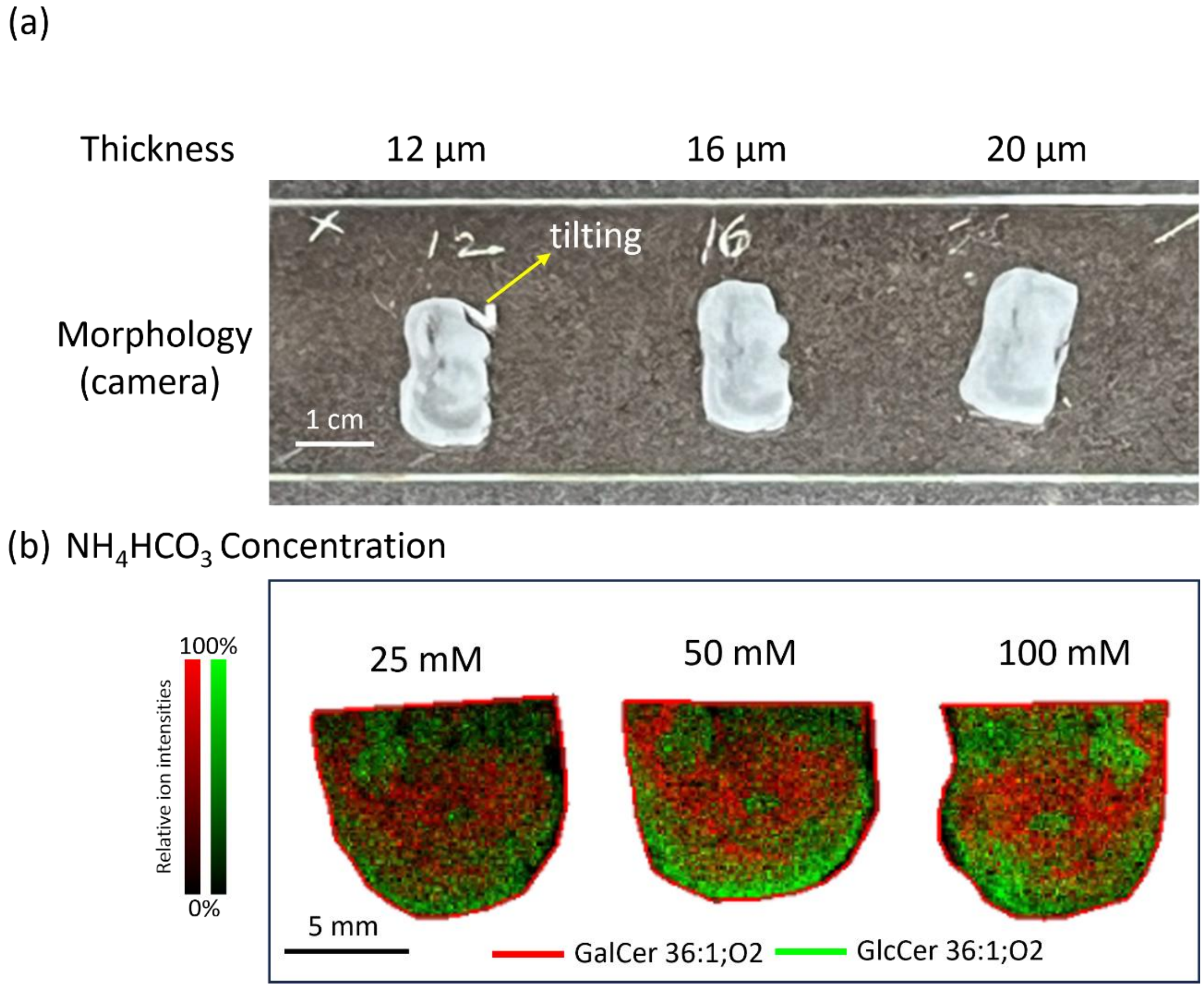

Fig. S8. Additional results from the optimization of sample preparation and washing. (a) Comparison of tissue thicknesses, showing that 12 μm sections are prone to morphological disruption after enzymatic treatment and washing. (b) Comparison of ammonium bicarbonate concentrations, showing marginal signal enhancement between 50 and 100 mM, with both conditions yielding higher signals than 25 mM.

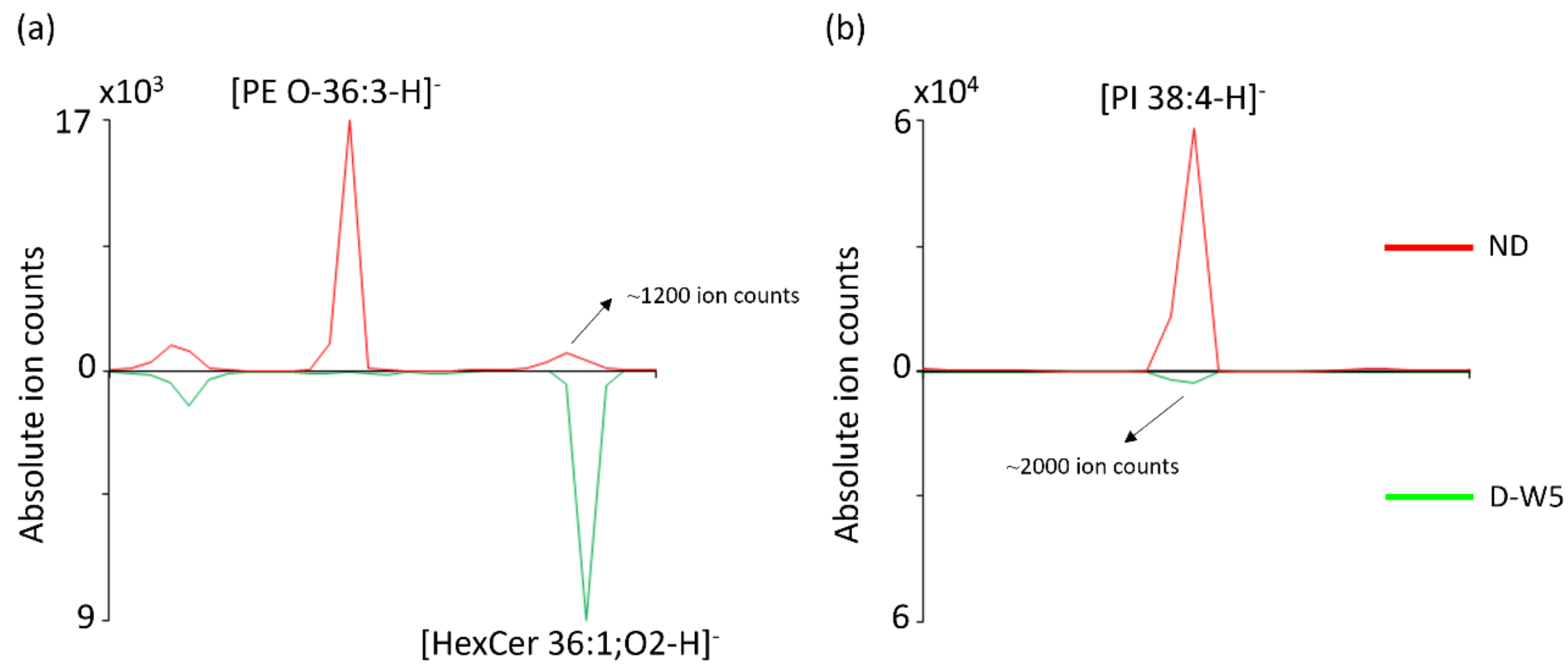

Fig. S9. Representative zoomed-in m/z spectra before and after PLC treatment with a washing step. (a) m/z 726.5–726.6, showing signal comparison between isobaric lipid species [PE O-36:3–H]⁻ (m/z 726.544) and [HexCer 36:1;O2–H]⁻ (m/z 726.588). (b) m/z 885.5–885.6, showing signal reduction of the abundant phospholipid [PI 38:4–H]⁻ (m/z 885.546). ND: no PLC digestion/treatment; D-W5 with PLC digestion/treatment followed by 5 min washing steps.

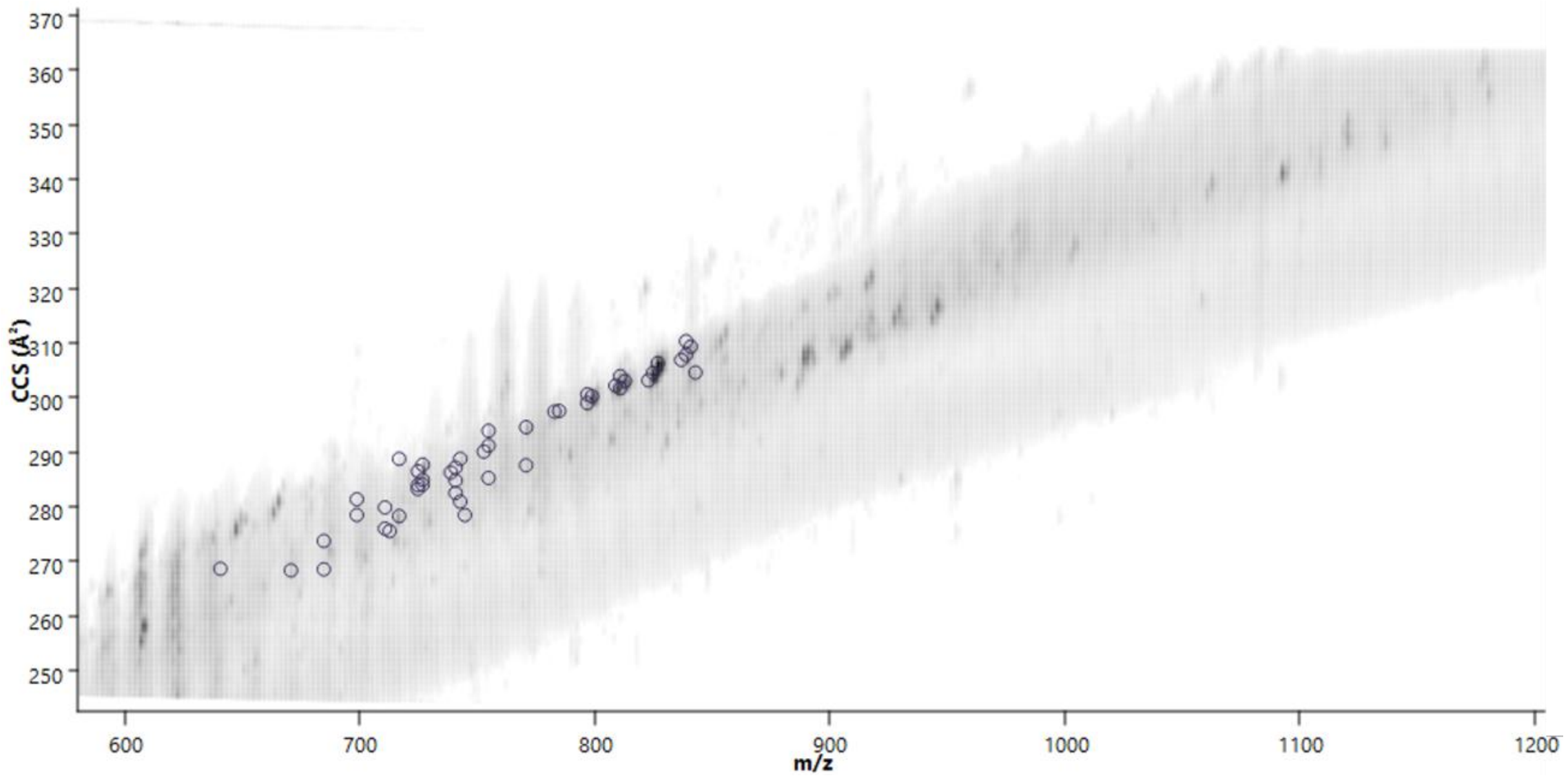

Fig. S10. 2D ion mobility–m/z spectrum with extracted cerebroside (CB) species highlighted by circles.

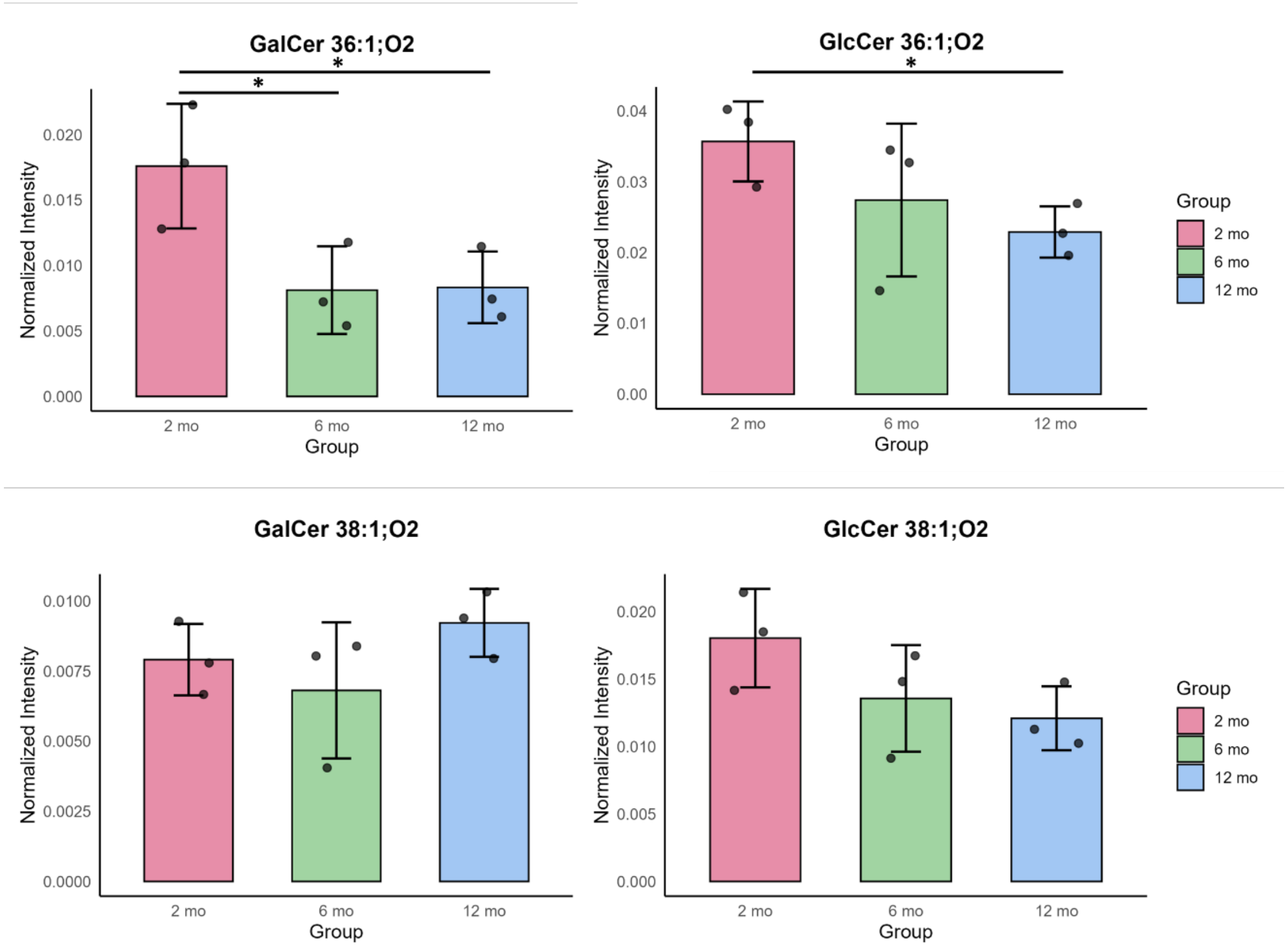

Fig. S11. Bar plots of representative isomeric pairs, GalCer 36:1;O2 vs. GlcCer 36:1;O3 and GalCer 38:1;O2 vs. GlcCer 38:1;O2, across ages (2 mo, 6 mo, and 12 mo) from biological replicates (n = 3). Each species was normalized to the total ion signal. Each species was normalized to the total ion signal. Error bars represent standard deviations. $p < 0.05$ (Games–Howell test)

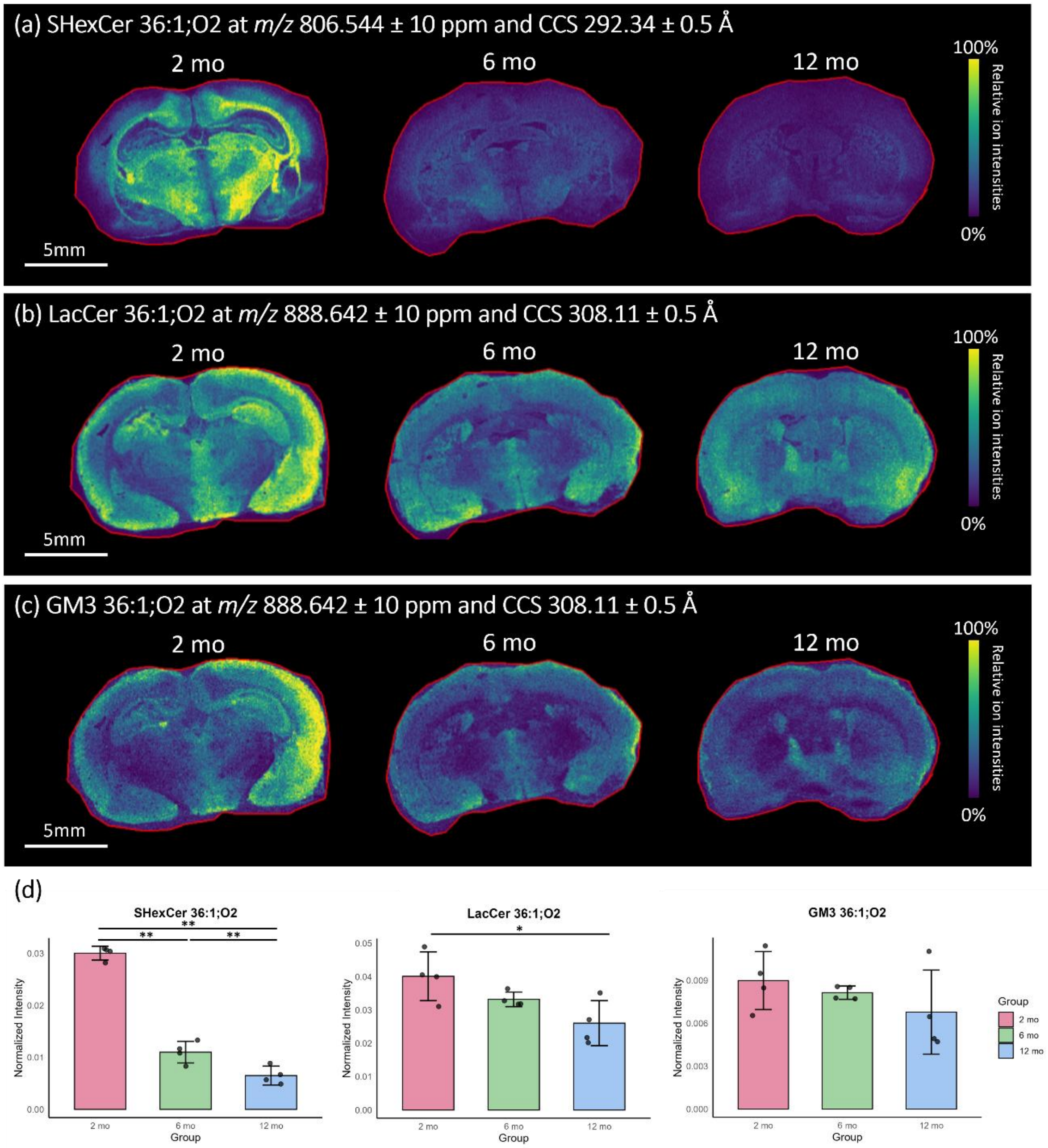

Fig. S12. Downstream glycolipids from the de novo metabolic pathway to isomeric cerebrosides. Ion images from three age groups (2 mo, 6 mo, and 12 mo) of (a) SHexCer 36:1;O2, (b) LacCer 36:1;O2, and (c) GM3 36:1;O2. (d) Bar plots showing age-dependent changes in downstream glycolipids. Each species was normalized to the total ion signal. Error bars represent standard deviations. $p < 0.05$ and *$p < 0.01$ (Games–Howell test).

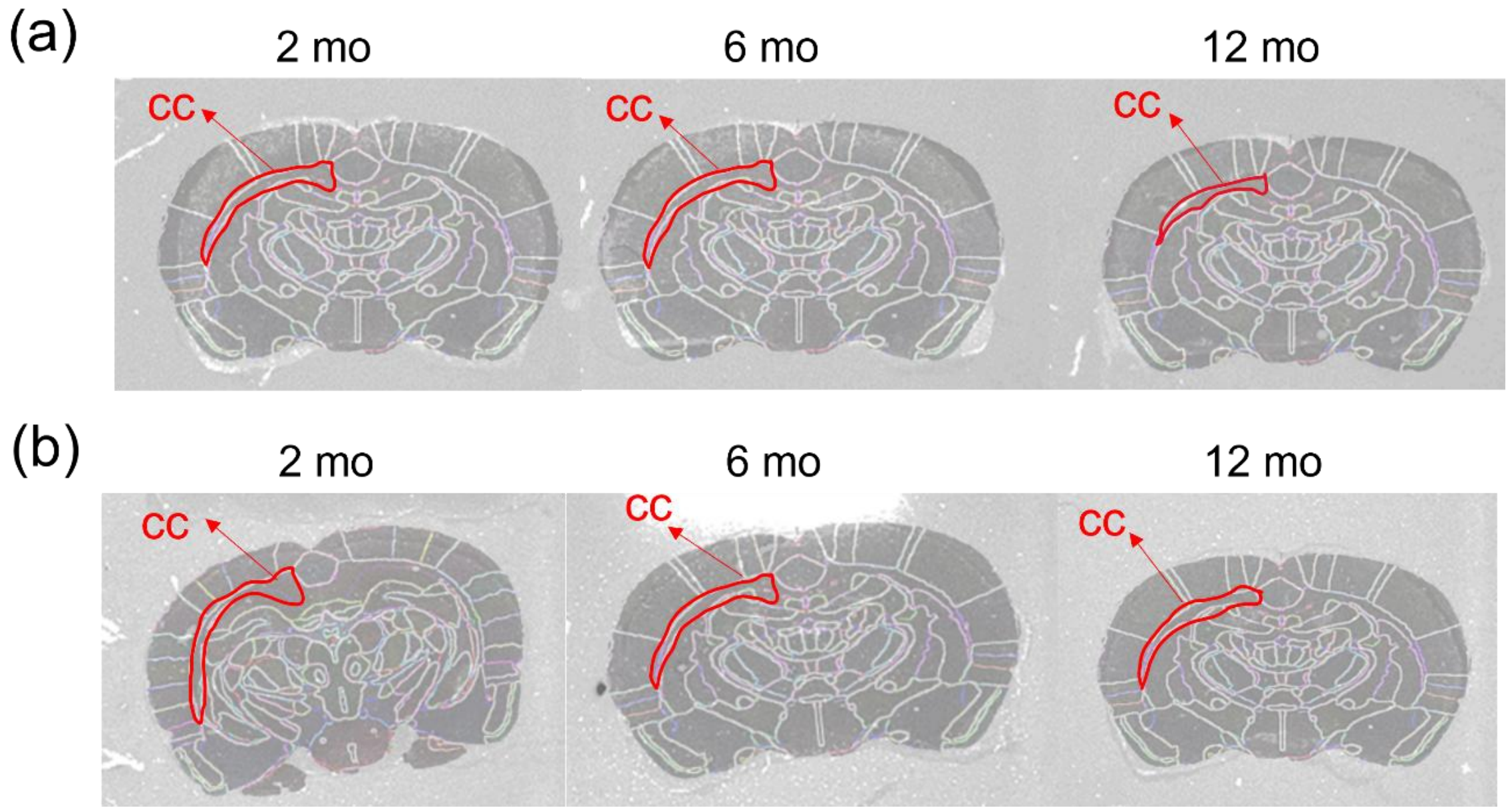

Fig. S13. Overlaid optical images and corresponding rat brain atlas sections for (a) lower-resolution MSI acquisitions used for statistical analysis and (b) higher-resolution MSI acquisitions used for Figure 5. Due to visual limitations in labeling every anatomical region, only the corpus callosum (cc) and the regions selected for regional analysis are labeled in the left hemisphere. For panel (a), tissue sections correspond to the atlas navigation coordinates (x, y, z) = (0.624, -3.612, 2.409) mm. In panel (b), samples from the 6- and 12-month age groups were sectioned to the atlas navigation coordinates (x, y, z) = (-0.076, -1.547, 2.409) mm because tissue from these animals had been exhausted during statistical imaging experiments. Atlas images were adapted from the Waxholm Space Atlas of the Sprague Dawley Rat Brain Online Library.[1] Additional atlas references and detailed regional classifications are available through EBRAINS (https://ebrains.eu/data-tools-services/brain-atlases/rat-brain).

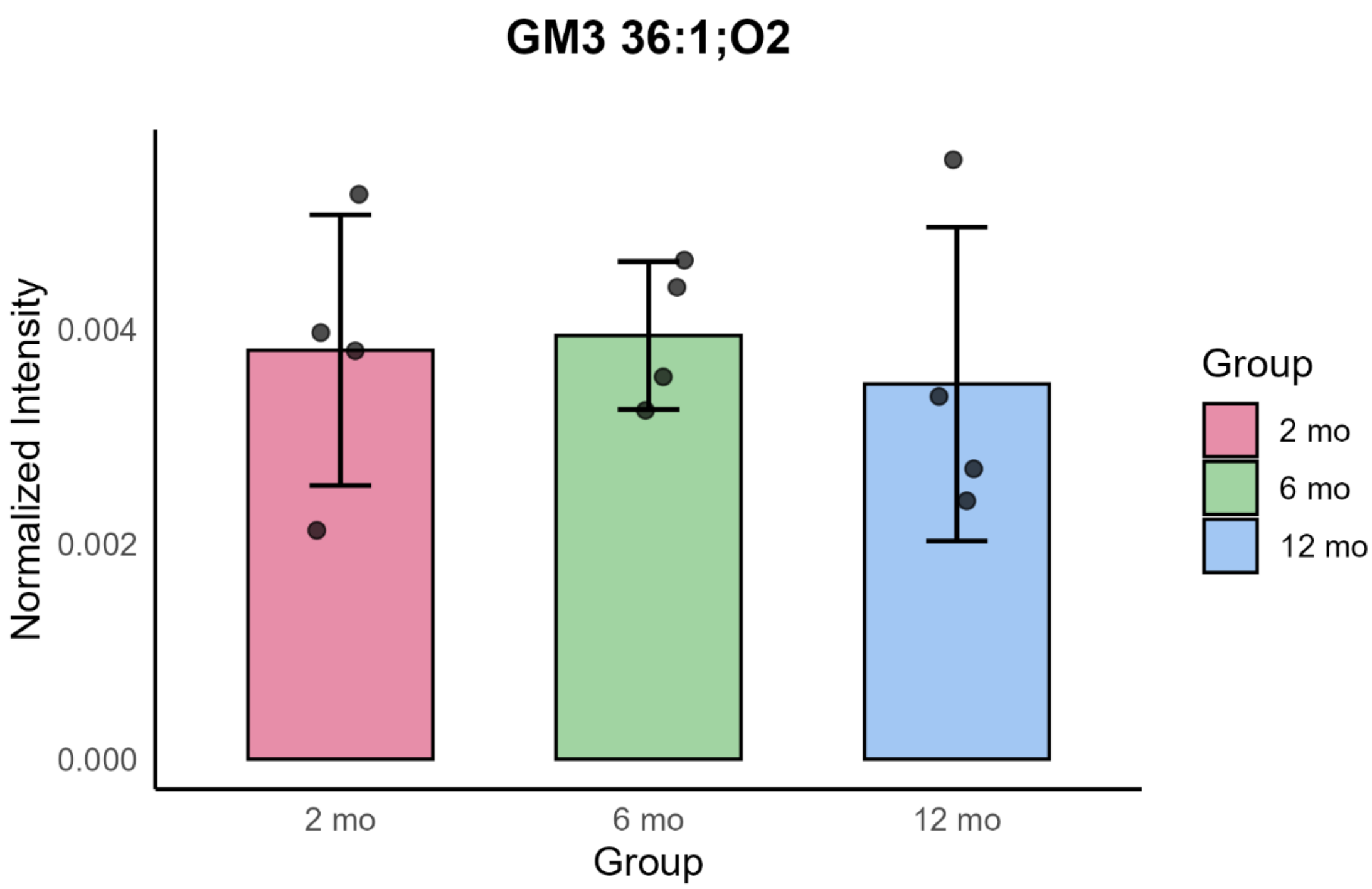

Fig. S14. Comparison of GM3 36:1;O2 in the cc region, highlighted by the white contour in Figure 5a (representative regions). Each species was normalized to the total ion signal. Error bars represent standard deviations.

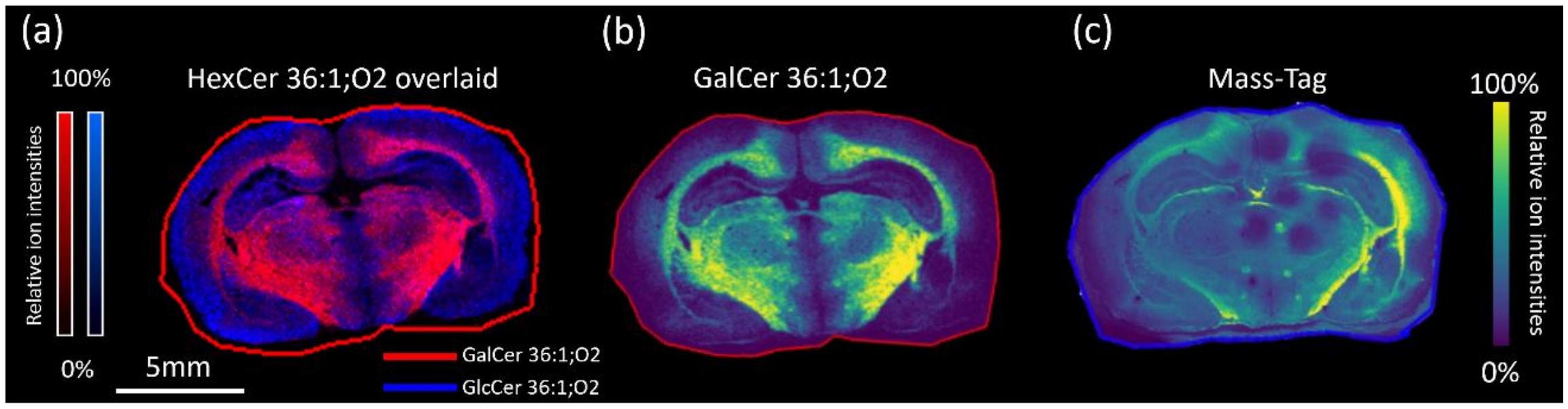

Fig. S15. Ion images of HexCer 36:1;O2 and a mass-tag target for myelin-associated proteins, demonstrating strong co-localization of GalCer with myelination-rich regions, likely associated with oligodendrocytes. (a) Overlay of isomeric cerebrosides HexCer 36:1;O2, (b) GalCer 36:1;O2 ion image, and (c) mass-tag image (m/z 1365.72). The same tissue section was first imaged using PLC-MALDI-TIMS MSI and subsequently analyzed by mass-tag MSI, resulting in aliquot interference in (c); however, higher abundance is still observed around the corpus callosum region.

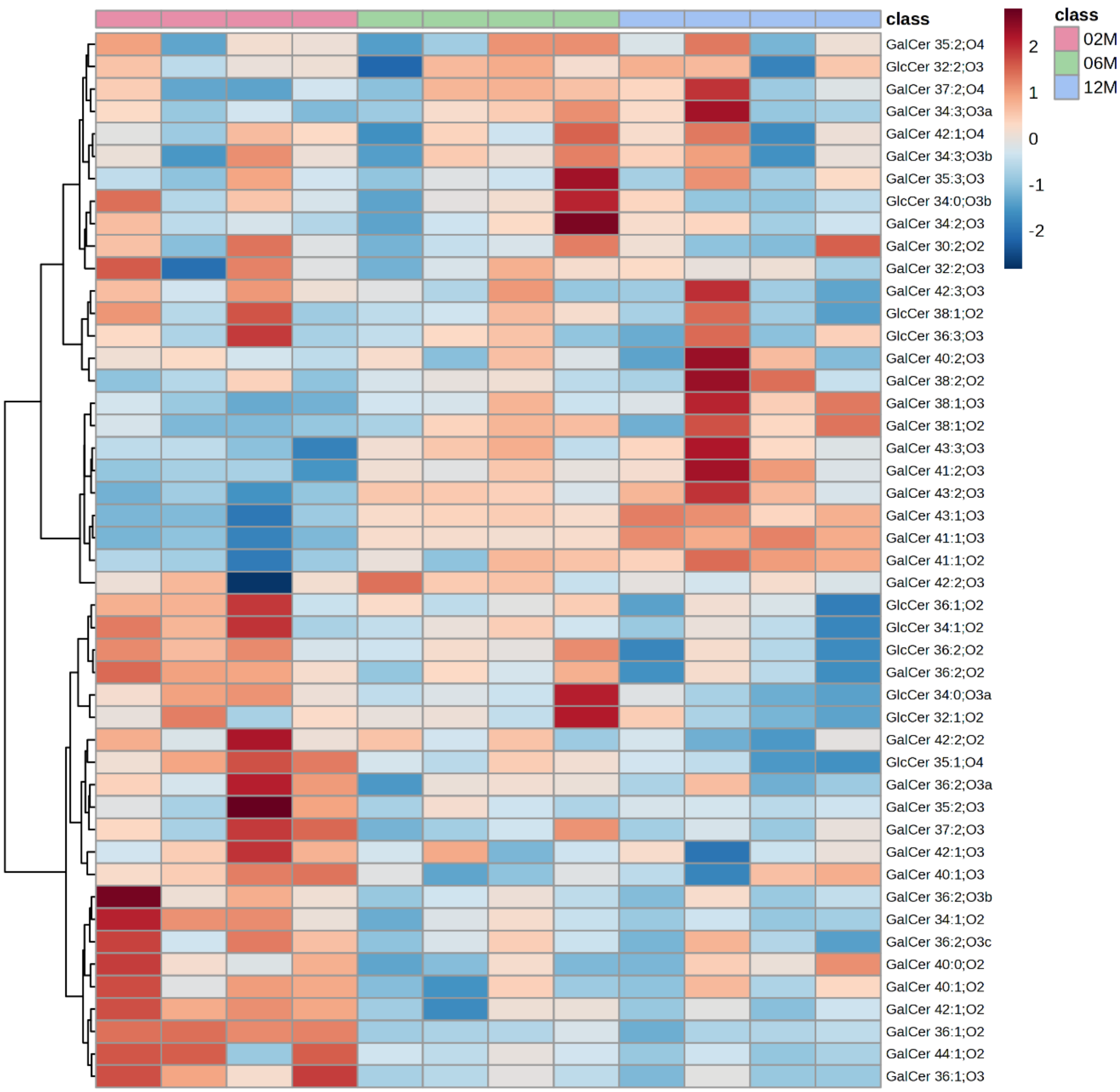

Fig. S16. Enlarged heatmap of global cerebroside profiling results in rat brain among three age groups, 2 mo, 6 mo, and 12 mo (from Figure 4a).

Tables

Table S1. Abbreviations

Abbreviation	Full name	Abbreviation	Full name
**GalCer**	galactosylceramide	**GlcCer**	glucosylceramide
**CB or HexCer**	Cerebroside or Hexosylceramides	**LacCer or Hex_2Cer**	lactosylceramde
**SHexCer**	sulfatides	**GM3**	Ganglioside M3
**MSI**	Mass spectrometry imaging	**IMS**	Ion mobility spectrometry
**PLC**	phospholipase C	**TIMS**	trapped ion mobility spectrometry
**SLIMs**	structures for lossless ion manipulations	**TWIMS**	traveling wave IMS
**cIMS**	cyclic IMS	**EIM**	Extracted ion mobility distributions
**PE**	Phosphatidylethanolamine	**PI**	Phosphatidylinositol
**cc**	corpus callosum	**ht**	hypothalamus
**GALC**	galactosylceramidase	**CGT**	UDP-galactose-ceramide galactosyltransferase
**GCS**	UDP-glucose-ceramide glucosyltransferase	**GLUC**	glucosylceramidase
**CST**	cerebroside sulfotransferase	**ASA**	arylsulfatase A
**GALT**	β-1,4-galactosyltransferase 5/6	**GBA**	glucocerebrosidase
**ST3GAL5**	GM3 synthase (gene: ST3GAL5)	**Neu**	sialidase
**PCA**	Principal Component Analysis	**ANOVA**	Analysis of variance
**FDR**	False Discovery Rate	**CerS**	Ceramide synthase
**Cgt**	Gene for UDP-galactose-ceramide galactosyltransferase (CGT)	**GLTP**	glycolipid transfer protein
Gltp	Gene for glycolipid transfer protein	**ER**	endoplasmic reticulum
ITO	Indium tin oxide	**DAN**	1,5-Diaminonaphthalene
**ACN**	Acetonitrile		

Table S2. Calculated FWHM resolving power (Rp) values for GlcCer and GalCer.

**Rp from different Pressure**		
**Pressure (mbar)**	**GlcCer**	**GalCer**
1.8	120	120
2.0	130	140
2.2	140	140
2.4	140	140
2.6	140	150
2.8	150	150
**Rp from different Ramp Time**		
**Ramp Time (ms)**	**GlcCer**	**GalCer**
100	NA	70
200	130	130
300	130	140
500	140	140
600	140	150
800	150	150
1000	150	160
1500	150	160
1800	160	170

*Calculated from Figure 1b and Figure S4 and S5. At a ramp time of 100 ms, the two mobility peaks substantially overlapped; therefore, only the resolution of the first peak (GalCer) is reported. Values were rounded to the nearest 10.

Table S3. Detailed optimized instrumental settings.

**Laser settings**	
Parameter	Setting
Shots per pixel	50
Laser frequency	1 kHz
Laser power	70%
Power boost	5%
MALDI-2 lase pulse time	5µs
**Ion optics and ion transfer (general)**	
Scan Range	400 – 2000 (m/z)
Deflection 1 Delta	-20.0V
Funnel 1 RF	250 Vpp
Funnel 2 RF	250 Vpp
Collision RF	1800 Vpp
Collisional energy	10 eV
Transfer time	65µs
Pre-pulse storage	5 µs
**TIMS-related settings**	
t_{accum}	51 ms (related to shots/pixel)
t_{ramp}	800 ms
$1/k_o$ ranges (V s cm^{-2})	1.20 – 1.80
imex parameters	
Δt1	20 V
Δt2	20 V
Δt3	-50 V
Δt4	-50 V
Δt5	0 V
Δt6	-100 V
Collision Cell In	-300 V
**Mass-tag analysis instrument settings**	
Scan Range	800 - 2500
Polarity	positive
Shots per pixel	500
Laser frequency	10 kHz
Laser power	50%
Lase size	50 µm
Deflection 1 Delta	70 V
Funnel 1 RF	450 Vpp
Funnel 2 RF	400 Vpp
Collision RF	2500 Vpp
Collisional energy	10 eV
Transfer time	90 µs
Pre-pulse storage	7 µs

Table S4. Signal enhancement factors derived from Figure 2.

Species	observed m/z	Enhancement factor
HexCer 34:1;O2	698.5585	3.5
HexCer 36:2;O2	724.5739	7.4
HexCer 36:1;O2	726.5874	14.3
HexCer 36:2;O3	740.5690	7.1
HexCer 38:1;O2	754.6197	6.2
GlcCer 30:2:O2	640.4788$^{\$}$	15.1
GlcCer 32:1;O2	670.5257$^{\$}$	4.9

The signal enhancement factor was calculated from the averaged ion intensities across the whole ion image using the tissue shown in Figure 2. The comparison was performed between the ND w/o M2 group and the D-5W with M2 group. $^{\$}$The two GlcCer species showed better mass accuracy in these normal brain tissues than in our biological models.

Table S5. Profiled isomeric cerebrosides in aged rat brains

Species	Theoretical m/z	Measured m/z*	Mass accuracy**	1/k0***	CCS (Å)*
GalCer 30:2;O2	640.4788	640.4859 ± 0.0027	11.2$	1.312	268.37 ± 0.36
GlcCer 32:1;O2	670.5258	670.5180 ± 0.0029	-11.7$	1.310	267.78 ± 0.41
GalCer 32:2;O3	684.5051	684.5043 ± 0.0011	-1.2	1.312	268.00 ± 0.44
GlcCer 32:2;O3	684.5051	684.5043 ± 0.0011	-1.2	1.338	273.19 ± 0.42
GalCer 34:1;O2	698.5571	698.5589 ± 0.0013	2.6	1.362	277.98 ± 0.47
GlcCer 34:1;O2	698.5571	698.5589 ± 0.0013	2.6	1.374	280.51 ± 0.66
GalCer 34:3;O3[a]	710.5207	710.5211 ± 0.0002	0.6	1.355	275.59 ± 0.50
GalCer 34:3;O3[b]	710.5207	710.5211 ± 0.0002	0.6	1.364	279.32 ± 0.30
GalCer 34:2;O3	712.5364	712.5351 ± 0.0005	-1.9	1.346	274.70 ± 0.27
GlcCer 34:0;O3[a]	716.5677	716.5779 ± 0.0038	14.3$	1.367	277.74 ± 0.33
GlcCer 34:0;O3[b]	716.5677	716.5779 ± 0.0038	14.3$	1.405	288.15 ± 0.27
GalCer 35:3;O3	724.5364	724.5343 ± 0.0008	-2.9	1.385	282.55 ± 0.28
GalCer 36:2;O2	724.5727	724.5734 ± 0.0003	1.0	1.389	283.39 ± 0.36
GlcCer 36:2;O2	724.5727	724.5734 ± 0.0003	1.0	1.400	285.75 ± 0.39
GalCer 35:2;O3	726.552	726.5513 ± 0.0003	-1.0	1.389	283.36 ± 0.42
GalCer 36:1;O2	726.5884	726.5875 ± 0.0003	-1.4	1.395	284.52 ± 0.45
GlcCer 36:1;O2	726.5884	726.5875 ± 0.0003	-1.4	1.408	287.18 ± 0.45
GlcCer 36:3;O3	738.552	738.5503 ± 0.0007	-2.2	1.402	285.46 ± 1.57
GalCer 36:2;O3[a]	740.5677	740.5688 ± 0.0004	1.5	1.389	281.97 ± 0.42
GalCer 36:2;O3[b]	740.5677	740.5688 ± 0.0004	1.5	1.400	284.33 ± 0.44
GalCer 36:2;O3[c]	740.5677	740.5688 ± 0.0004	1.5	1.395	286.67 ± 0.44
GalCer 35:2;O4	742.5469	742.5466 ± 0.0015	-0.4	1.374	280.26 ± 0.43
GalCer 36:1;O3	742.5833	742.5840 ± 0.0016	1.0	1.412	288.09 ± 0.34
GlcCer 35:1;O4	744.5626	744.5554 ± 0.0031	-9.6$	1.363	277.96 ± 0.45
GalCer 38:2;O2	752.5677	752.5963 ± 0.0108	38.0$$	1.422	290.00 ± 0.06

GalCer 37:2;O3	754.5833	754.5825 ± 0.0006	-1.1	1.397	284.78 ± 0.39
GalCer 38:1;O2	754.6197	754.6195 ± 0.0002	0.3	1.429	290.65 ± 0.38
GlcCer 38:1;O2	754.6197	754.6195 ± 0.0002	0.3	1.436	293.44 ± 0.40
GalCer 37:2;O4	770.5782	770.5768 ± 0.0006	-1.8	1.408	286.96 ± 0.48
GalCer 38:1;O3	770.6160	770.6146 ± 0.0018	-1.8	1.443	294.11 ± 0.41
GalCer 40:1;O2	782.651	782.6520 ± 0.0013	1.3	1.458	297.11 ± 0.48
GalCer 40:0;O2	784.6666	784.6588 ± 0.0030	9.9$	1.458	297.13 ± 0.40
GalCer 40:2;O3	796.6303	796.6298 ± 0.0014	0.6	1.464	298.13 ± 0.41
GalCer 41:1;O2	796.6666	796.6683 ± 0.0006	2.1	1.473	300.07 ± 0.49
GalCer 40:1;O3	798.6459	798.6467 ± 0.0003	1.0	1.471	299.69 ± 0.43
GalCer 42:2;O2	808.6666	808.6680 ± 0.0005	1.7	1.481	301.63 ± 0.33
GalCer 41:2;O3	810.6459	810.6442 ± 0.0007	-2.1	1.479	301.25 ± 0.59
GalCer 42:1;O2	810.6823	810.6841 ± 0.0007	2.3	1.490	303.34 ± 0.42
GalCer 41:1;O3	812.6616	812.6609 ± 0.0004	-0.8	1.485	302.50 ± 0.41
GalCer 42:3;O3	822.6459	822.6472 ± 0.0017	1.6	1.486	302.61 ± 0.50
GalCer 42:2;O3	824.6616	824.6615 ± 0.0002	-0.1	1.492	303.76 ± 0.36
GalCer 42:1;O3	826.6772	826.6790 ± 0.0019	2.2	1.502	305.75 ± 0.50
GalCer 43:3;O3	836.6616	836.6605 ± 0.0005	-1.3	1.504	306.15 ± 0.59
GalCer 43:2;O3	838.6772	838.6769 ± 0.0016	-0.4	1.510	307.24 ± 0.44
GalCer 44:1;O2	838.7136	838.7133 ± 0.0004	-0.4	1.522	309.89 ± 0.55
GalCer 43:1;O3	840.6929	840.6935 ± 0.0020	0.7	1.517	308.70 ± 0.28
GalCer 42:1;O4	842.6721	842.6673 ± 0.0021	-5.7	1.493	303.86 ± 0.53

*m/z and CCS values are reported as mean ± SD from two sample sets; CCS calibration was performed using voltages and a tuning mix on the TIMS system (see Methods for details). **Mass accuracies are calculated from the mean m/z values. ***1/k_0 values are reported as mean; the same calibration as CCS was applied. $Glycolipids with lipid composition were near the detection limit, which may contribute to slightly higher ppm error. $$ GalCer 38:2;O2 showed high variation across different samples, leading to reduced mass accuracy; but we further confirmed using additional fresh rat brain, and it was not eliminated after enzymatic digestion. a,b,cletters annotated potential different lipid compositions (e.g., fatty acyl chains)

Table S6. Highlighted isomeric cerebrosides in aged rat brains.

**Lipid composition**	**Theoretical *m/z***	**Measured *m/z****	**Mass accuracy****	**CCS (Å)*****	**Species**
32:2;O3	684.5051	684.5043 ± 0.0011	-1.2 ppm	268.00 ± 0.44	GalCer
				273.19 ± 0.42	GlcCer
34:1;O2	698.5571	698.5589 ± 0.0013	2.6 ppm	277.98 ± 0.47	GalCer
				280.51 ± 0.66	GlcCer
34:3;O3	710.5207	710.5211 ± 0.0002	0.6 ppm	275.59 ± 0.50	GalCer[a]
				279.32 ± 0.30	GalCer[b]
34:0;O3	716.5677	716.5780 ± 0.0038	14.3 ppm$	277.74 ± 0.33	GlcCer[a]
				288.15 ± 0.27	GlcCer[b]
36:2;O2	724.5727	724.5734 ± 0.0003	1.0 ppm	283.39 ± 0.36	GalCer
				285.75 ± 0.39	GlcCer
36:1;O2	726.5884	724.5875 ± 0.0003	- 1.4 ppm	284.52 ± 0.45	GalCer
				287.18 ± 0.45	GlcCer
36:2;O3	740.5677	740.5688 ± 0.0004	1.5 ppm	281.97 ± 0.42	GalCer[a]
				284.33 ± 0.44	GalCer[b]
				286.67 ± 0.44	GalCer[c]
38:1;O2	754.6197	754.6195 ± 0.0002	0.3 ppm	290.65 ± 0.38	GalCer
				293.44 ± 0.40	GlcCer

**m/z* values are reported as mean ± SD from two sample sets. **Mass accuracies are calculated from the mean m/z values. ***CCS values are reported as mean ± SD from two sample sets; CCS calibration was performed using voltages and a tuning mix on the TIMS system (see Methods for details). $Glycolipids with lipid composition 34:0;O3 were near the detection limit, which may contribute to slightly higher ppm error. a,b,cletters annotated potential different lipid compositions (e.g., fatty acyl chains)

Table S7. Loading scores of the first two principal components from PCA.

**Species**	**PC1**	**PC2**
GalCer 30:2;O2	-0.00512	-3.92E-05
GlcCer 32:1;O2	-0.00423	0.007077
GalCer 32:2;O3	-0.00116	0.004486
GlcCer 32:2;O3	-0.00017	0.003782
GalCer 34:1;O2	-0.00487	0.011581
GlcCer 34:1;O2	-0.00174	0.004183
GalCer 34:3;O3a	0.002069	0.003463
GalCer 34:3;O3b	-0.00082	0.001945
GalCer 34:2;O3	0.00156	0.006963
GlcCer 34:0;O3[a]	-0.04559	0.051787
GlcCer 34:0;O3[b]	-0.01868	0.031193
GalCer 35:3;O3	-0.00023	0.004889
GalCer 36:2;O2	-0.00277	0.00865
GlcCer 36:2;O2	-0.00446	0.015531
GalCer 35:2;O3	-0.01013	0.002383
GalCer 36:1;O2	-0.09246	0.14063
GlcCer 36:1;O2	-0.07666	0.20194
GlcCer 36:3;O3	-0.00051	0.002545
GalCer 36:2;O3[a]	-0.00788	0.013522
GalCer 36:2;O3[b]	-0.00331	0.013929
GalCer 36:2;O3[c]	-0.0027	0.011907
GalCer 35:2;O4	0.008245	0.054963
GalCer 36:1;O3	-0.02667	0.080837
GlcCer 35:1;O4	-0.01659	0.023974
GalCer 38:2;O2	0.004731	0.002712
GalCer 37:2;O3	-0.00644	0.007426
GalCer 38:1;O2	0.05466	0.020873
GlcCer 38:1;O2	-0.00905	0.10135
GalCer 37:2;O4	0.029028	0.022739
GalCer 38:1;O3	0.097269	0.016622
GalCer 40:1;O2	-0.019	0.10233
GalCer 40:0;O2	-0.00059	0.008451
GalCer 40:2;O3	0.036782	0.10142
GalCer 41:1;O2	0.0517	-0.0057
GalCer 40:1;O3	-0.18642	-0.03207
GalCer 42:2;O2	-0.13381	0.10163
GalCer 41:2;O3	0.12373	0.005074
GalCer 42:1;O2	-0.0768	0.24097
GalCer 41:1;O3	0.50217	-0.64031
GalCer 42:3;O3	0.002153	0.11604
GalCer 42:2;O3	0.28955	-0.12497

GalCer 42:1;O3	-0.72118	-0.60427
GalCer 43:3;O3	0.030073	0.002397
GalCer 43:2;O3	0.14547	-0.07393
GalCer 44:1;O2	-0.00518	0.024827
GalCer 43:1;O3	0.10904	-0.10998
GalCer 42:1;O4	-0.00304	0.014253

Table S8. The statistic results of isomeric cerebrosides among three aged groups, 2 months (2 mo), 6 months (6 mo), and 12 months (12 mo) (n=4).

Species	F-score*	p-values**				FDR_Adjusted***			
		Welch's	2 mo vs 6 mo	2 mo vs 12 mo	6 mo vs 12 mo	Welch's	2 mo vs 6 mo	2 mo vs 12 mo	6 mo vs 12 mo
GalCer 30:2;O2	0.64	0.552	0.320	0.394	0.998	0.692	0.574	0.617	0.998
GalCer 32:2;O3	1.43	0.289	0.345	0.195	0.569	0.424	0.591	0.412	0.764
GalCer 34:1;O2	11.26	0.004	0.036	0.020	0.177	0.024	0.164	0.133	0.397
GalCer 34:2;O3	0.61	0.565	0.463	0.961	0.467	0.692	0.672	0.990	0.672
GalCer 34:3;O3[a]	0.56	0.589	0.112	0.581	0.785	0.692	0.315	0.772	0.895
GalCer 34:3;O3[b]	0.15	0.865	0.750	0.834	0.622	0.924	0.881	0.941	0.784
GalCer 35:2;O3	3.25	0.087	0.162	0.142	0.732	0.163	0.385	0.358	0.875
GalCer 35:2;O4	0.01	0.994	0.962	0.905	0.960	0.994	0.990	0.960	0.990
GalCer 35:3;O3	0.23	0.796	0.623	0.912	0.601	0.870	0.784	0.960	0.778
GalCer 36:1;O2	79.56	<0.001	<0.001	<0.001	0.322	<0.001	0.014	0.016	0.574
GalCer 36:1;O3	11.89	0.003	0.020	0.013	0.533	0.024	0.133	0.133	0.747
GalCer 36:2;O2	4.09	0.055	0.101	0.053	0.295	0.121	0.297	0.189	0.548
GalCer 36:2;O3[a]	8.35	0.009	0.034	0.019	0.463	0.047	0.164	0.133	0.672
GalCer 36:2;O3[b]	6.32	0.019	0.069	0.048	0.590	0.076	0.221	0.178	0.777
GalCer 36:2;O3[c]	6.12	0.021	0.015	0.033	0.520	0.076	0.133	0.164	0.741
GalCer 37:2;O3	4.02	0.057	0.112	0.037	0.600	0.121	0.315	0.164	0.778
GalCer 37:2;O4	1.80	0.220	0.122	0.256	0.844	0.370	0.325	0.501	0.945
GalCer 38:1;O2	3.67	0.068	0.003	0.114	0.746	0.134	0.042	0.315	0.881
GalCer 38:1;O3	6.60	0.017	0.048	0.023	0.184	0.074	0.178	0.138	0.405
GalCer 38:2;O2	1.64	0.246	0.441	0.196	0.330	0.386	0.662	0.412	0.575

GalCer 40:0;O2	2.35	0.151	0.058	0.352	0.326	0.266	0.200	0.591	0.574
GalCer 40:1;O2	5.23	0.031	0.034	0.047	0.432	0.086	0.164	0.178	0.655
GalCer 40:1;O3	1.51	0.271	0.190	0.149	0.994	0.411	0.411	0.368	0.998
GalCer 40:2;O3	0.03	0.966	0.997	0.853	0.860	0.987	0.998	0.947	0.948
GalCer 41:1;O2	10.64	0.004	0.046	0.002	0.164	0.025	0.178	0.039	0.385
GalCer 41:1;O3	46.41	<0.001	0.001	<0.001	0.015	<0.001	0.025	0.009	0.133
GalCer 41:2;O3	6.80	0.016	0.004	0.041	0.365	0.074	0.050	0.169	0.592
GalCer 42:1;O2	11.49	0.003	0.021	0.001	0.972	0.024	0.133	0.019	0.993
GalCer 42:1;O3	1.37	0.303	0.312	0.155	0.611	0.432	0.571	0.376	0.784
GalCer 42:1;O4	0.28	0.761	0.674	0.713	0.551	0.851	0.819	0.860	0.754
GalCer 42:2;O2	0.67	0.537	0.416	0.407	0.911	0.692	0.638	0.631	0.960
GalCer 42:2;O3	0.58	0.579	0.382	0.547	0.649	0.692	0.605	0.754	0.796
GalCer 42:3;O3	0.44	0.657	0.265	0.535	0.904	0.754	0.511	0.747	0.960
GalCer 43:1;O3	25.26	<0.001	0.004	<0.001	0.054	0.003	0.050	0.016	0.189
GalCer 43:2;O3	11.25	0.004	0.002	0.015	0.365	0.024	0.039	0.133	0.592
GalCer 43:3;O3	5.33	0.030	0.019	0.038	0.642	0.086	0.133	0.164	0.796
GalCer 44:1;O2	5.68	0.025	0.134	0.068	0.038	0.085	0.349	0.221	0.164
GlcCer 32:1;O2	1.03	0.395	0.775	0.208	0.293	0.530	0.895	0.425	0.548
GlcCer 32:2;O3	4.05	0.056	0.142	0.030	0.467	0.121	0.358	0.164	0.672
GlcCer 34:0;O3[a]	2.33	0.153	0.771	0.016	0.224	0.266	0.895	0.133	0.445
GlcCer 34:0;O3[b]	1.74	0.230	0.873	0.098	0.200	0.373	0.954	0.293	0.415
GlcCer 34:1;O2	3.77	0.065	0.215	0.066	0.176	0.132	0.434	0.221	0.397
GlcCer 35:1;O4	5.09	0.033	0.095	0.014	0.357	0.087	0.292	0.133	0.591
GlcCer 36:1;O2	5.23	0.031	0.176	0.033	0.117	0.086	0.397	0.164	0.317
GlcCer 36:2;O2	4.87	0.037	0.350	0.038	0.079	0.091	0.591	0.164	0.247

GlcCer 36:3;O3	0.11	0.898	0.644	0.787	0.890	0.938	0.796	0.895	0.960
GlcCer 38:1;O2	1.04	0.393	0.380	0.273	0.569	0.530	0.605	0.519	0.764

*All values are rounded to the reported digits, including F-statistics, p-values, and FDR-adjusted p-values. **p-values include results from Welch's ANOVA across all three groups as well as pairwise comparisons using the Games–Howell t-test. ***FDR-adjusted values reflect corrections applied to both Welch's ANOVA and pairwise t-tests using the Benjamini–Hochberg procedure.

Table S9. The statistic results from pooled cerebrosides.

Species	F-score*	p-values**				FDR_Adjusted***			
		Welch's	2 mo vs 6 mo	2 mo vs 12 mo	6 mo vs 12 mo	Welch's	2 mo vs 6 mo	2 mo vs 12 mo	6 mo vs 12 mo
HexCer 30:2;O2	0.64	0.552	0.320	0.394	0.998	0.759	0.467	0.573	0.998
HexCer 32:1;O2	1.03	0.395	0.775	0.208	0.293	0.600	0.862	0.344	0.926
HexCer 32:2;O3	0.01	0.992	0.940	0.910	0.966	0.994	0.988	0.937	0.998
HexCer 34:0;O3	2.57	0.131	0.794	0.011	0.202	0.261	0.862	0.057	0.851
HexCer 34:1;O2	9.32	0.006	0.055	0.022	0.122	0.027	0.158	0.071	0.814
HexCer 34:2;O3	0.61	0.565	0.463	0.961	0.467	0.759	0.587	0.961	0.986
HexCer 34:3;O3	0.32	0.734	0.288	0.777	0.707	0.876	0.467	0.906	0.998
HexCer 35:1;O4	5.09	0.033	0.095	0.014	0.357	0.084	0.241	0.057	0.926
HexCer 35:2;O3	3.25	0.087	0.162	0.142	0.732	0.183	0.324	0.280	0.998
HexCer 35:2;O4	0.01	0.994	0.962	0.905	0.960	0.994	0.988	0.937	0.998
HexCer 35:3;O3	0.23	0.796	0.623	0.912	0.601	0.889	0.763	0.937	0.986
HexCer 36:1;O2	18.30	0.001	0.008	0.002	0.092	0.009	0.051	0.015	0.814
HexCer 36:1;O3	11.89	0.003	0.020	0.013	0.533	0.019	0.088	0.057	0.986
HexCer 36:2;O2	4.62	0.042	0.225	0.041	0.128	0.099	0.407	0.098	0.814
HexCer 36:2;O3	13.67	0.002	0.003	0.010	0.486	0.018	0.030	0.057	0.986
HexCer 36:3;O3	0.11	0.898	0.644	0.787	0.890	0.975	0.764	0.906	0.998
HexCer 37:2;O3	4.02	0.057	0.112	0.037	0.600	0.126	0.265	0.098	0.986
HexCer 37:2;O4	1.80	0.220	0.122	0.256	0.844	0.398	0.273	0.405	0.998
HexCer 38:1;O2	0.31	0.743	0.314	0.631	0.922	0.876	0.467	0.799	0.998
HexCer 38:1;O3	6.60	0.017	0.048	0.023	0.184	0.060	0.152	0.071	0.851
HexCer 38:2;O2	1.64	0.246	0.441	0.196	0.330	0.425	0.578	0.338	0.926
HexCer 40:0;O2	2.35	0.151	0.058	0.352	0.326	0.287	0.158	0.536	0.926
HexCer 40:1;O2	5.23	0.031	0.034	0.047	0.432	0.084	0.129	0.104	0.986
HexCer 40:1;O3	1.51	0.271	0.190	0.149	0.994	0.448	0.360	0.280	0.998
HexCer 40:2;O3	0.03	0.966	0.997	0.853	0.860	0.994	0.997	0.937	0.998
HexCer 41:1;O2	10.64	0.004	0.046	0.002	0.164	0.020	0.152	0.015	0.851

HexCer 41:1;O3	46.41	<0.001	0.001	<0.001	0.015	0.001	0.030	0.002	0.574
HexCer 41:2;O3	6.80	0.016	0.004	0.041	0.365	0.060	0.030	0.098	0.926
HexCer 42:1;O2	11.49	0.003	0.021	0.001	0.972	0.019	0.088	0.009	0.998
HexCer 42:1;O3	1.37	0.303	0.312	0.155	0.611	0.480	0.467	0.280	0.986
HexCer 42:1;O4	0.28	0.761	0.674	0.713	0.551	0.876	0.776	0.875	0.986
HexCer 42:2;O2	0.67	0.537	0.416	0.407	0.911	0.759	0.565	0.573	0.998
HexCer 42:2;O3	0.58	0.579	0.382	0.547	0.649	0.759	0.537	0.717	0.986
HexCer 42:3;O3	0.44	0.657	0.265	0.535	0.904	0.833	0.457	0.717	0.998
HexCer 43:1;O3	25.26	<0.001	0.004	<0.001	0.054	0.004	0.030	0.009	0.680
HexCer 43:2;O3	11.25	0.004	0.002	0.015	0.365	0.019	0.030	0.057	0.926
HexCer 43:3;O3	5.33	0.030	0.019	0.038	0.642	0.084	0.088	0.098	0.986
HexCer 44:1;O2	5.68	0.025	0.134	0.068	0.038	0.080	0.282	0.145	0.680

*All values are rounded to the reported digits, including F-statistics, p-values, and FDR-adjusted p-values. **p-values include results from Welch's ANOVA across all three groups as well as pairwise comparisons using the Games–Howell t-test. ***FDR-adjusted values reflect corrections applied to both Welch's ANOVA and pairwise t-tests using the Benjamini–Hochberg procedure.

Table S10. The statistic results of the downstream glycolipids. (n=4).

Species	p-values*		
	2 mo vs 6 mo	2 mo vs 12 mo	6 mo vs 12 mo
SHexCer 36:1;O2	0.651	0.101	0.478
LacCer 36:1;O2	0.003	0.001	0.006
GM3 36:1;O2	0.225	0.034	0.092

*All values are rounded to the reported digit.

Table S11. The statistic results from the regional analysis in the corpus callosum (n = 4).

Species	p-values*		
	2 mo vs 6 mo	2 mo vs 12 mo	6 mo vs 12 mo
GalCer →SHexCer metabolic pathway			
GalCer 36:1;O2	0.046	0.035	0.984
SHexCer 36:1;O2	0.019	0.001	0.208
GlcCer →LacCer → GM3 metabolic pathway			
GlcCer 36:1;O2	0.759	0.165	0.433
LacCer 36:1;O2	0.997	0.227	0.252
GM3 36:1;O2	0.985	0.926	0.854

*All values are rounded to the reported digit.

Table S12. Imaging acquisition order used to minimize artificial batch effects.

Slides #	Acquisition order intra-slides		
	**1**	**2**	**3**
**1**	A3-1	B2-1	C1-1
**2**	B1-1	A2-1	C3-1
**3**	C2-1	B3-1	A1-1
**4**	A1-2	B1-2	C1-2
**5**	B2-2	C2-2	A2-2
**6**	C3-2	A3-2	B3-2
	**Sagittal sections and the fourth sample**		
**7**	A-Sagittal		A4-1
**8**	B-Sagittal		B4-1
**9**	C-Sagittal		C4-1

A: 2-month (61 days) sample, B: 6-month (180 days) sample, C: 12-month (364 days) sample. Ax-y denotes individual samples, where x indicates the sample number within the age group and y indicates the slide number (1 or 2) of individual sample. Statistical results were generated using slide 1 for each sample (n = 4), while the biological replicates (second set) correspond to slide 2 and were included in statistical analysis of the second set.

Supporting Text

Materials and methods

***Chemicals**.* Lipid standards and total cerebrosides extract (porcine brain) were purchased from Avanti Polar Lipids, Inc (Alabaster, AL). 1,5-Diaminonaphthalene (DAN), 9-Aminoacridine (9-AA), MS-graded water, ethanol, acetone, α-cyano-4-hydroxycinnamic acid (CHCA) and Phospholipase C (PLC from B. cereus) were obtained from Sigma-Aldrich (St. Louis, MO), and LC- or MS-graded acetonitrile, chloroform, and ammonium bicarbonate were purchased from Fisher Scientific (Pittsburgh, PA.) PLC was dissolved in MS-graded water and aliquot at ~50 μL at 50 U mL^{-1} and stored at –20 °C for future usage.

***Animals*.** Male Sprague-Dawley rats of varying ages were obtained from Inotiv (West Lafayette, IN) and housed for ~30 days under a 12-h light/dark cycle with ad libitum food and water. Euthanasia was performed via gradual asphyxiation using CO_2, in accordance with protocol number, 23228, approved by the Illinois Institutional Animal Care and Use Committee (IACUC) and following ARRIVE guidelines.

***Sample preparation**.* Immediately following euthanasia, animals were transcardially perfused with 120 mL of ice-cold modified Gey's balanced salt solution (mGBSS).[2] Brains were rapidly dissected, flash-frozen on dry ice, and stored at –50 °C. For MSI analysis, brains were adhered to the cryostat-microtome (Leica Biosystems, Deer Park, IL) using Milli-Q water. Coronal and sagittal sections (16 μm thick) were cut and mounted onto ITO-coated glass slides (Delta Technologies, Loveland, CO). All brains were sectioned after minimal storage time and mounted onto glass slides, then stored at –20 °C prior to further preparation. Enzymatic treatment and matrix application were freshly performed before MSI acquisition. For enzymatic treatment, ~50 μL of a 50 U mL^{-1} PLC solution was evenly aliquoted onto each tissue surface and incubated at 37 °C for 30 mins (note that sagittal sections required slightly more than 50 μL due to their larger area.) After incubation, the surrounding solution was removed via vacuum suction, and the slides were air-dried. A gentle wash with 50 μL of 50 mM ammonium bicarbonate was performed for 5 mins. Isomeric standard mixture, GalCer 36:1;O2 and GlcCer 36:1;O2, at 50 μg mL^{-1} were spotted onto each slide for reference. DAN matrix (20 mg mL^{-1} in 70:30 (v/v) ACN/H_2O) was applied using the HTX M5 Sprayer (HTX Technologies, Chapel Hill, NC) under the following parameters: 4 passes, 100 μL min^{-1} flow rate, 60 °C nozzle temperature, 60 mm

spray distance, ~10 psi pressure, and 1500 mm min^{-1} spray velocity. Slides were stored briefly in a nitrogen chamber before MSI acquisition.

Spiking experiment. Mixtures of isomeric GalCer 34:1;O2 and GlcCer 34:1;O2 at two molar ratios (1:1 and 200:1) were spiked into a 1 mg mL^{-1} porcine brain extract. The resulting solution was mixed with a 20 mg mL^{-1} DAN matrix solution and spotted onto ITO-coated glass slides. The same MALDI-TIMS-MS parameters were applied to the two spots without the imaging workflow (i.e., without pixel-by-pixel acquisition). Results were extracted using Bruker Compass DataAnalysis (version 2025b, Bruker Daltonics, Bremen, Germany).

Mass-tag immunolabeling and MSI. Immunohistochemistry (IHC) of post-MSI tissue was performed using a photocleavable mass tag (PCMT) against myelin basic protein (MBP), following the MALDI HiPlex-IHC Miralys Imaging Laboratory Workflow (AmberGen, Billerica, MA) with minor modifications, including steps previously reported.3 Briefly, all steps specific to formalin-fixed paraffin-embedded (FFPE) tissue were omitted. Tissues were washed twice with cold acetone (−80 °C) for 3 min per wash, briefly dried using an N_2 gas line, and fixed with cold 4% paraformaldehyde (PFA) at 0 °C for 5 min. This was followed by a 10-min wash with 1× PBS, two separate acetone washes (3 min each), and a 3 min treatment with Carnoy's solution.

Rehydration was performed according to the MALDI HiPlex-IHC protocol, with ethanol treatments performed on ice (0 °C) to improve tissue morphology and preserve antigen structure. Additionally, we extended the cooling time after the antigen retrieval step to 1 h at room temperature (RT).

For each tissue section (roughly 1.5 cm^2), 70 µL of the working probe mixture containing 2.5 µg mL^{-1} MBP (AmberGen, Billerica, MA) was applied. Incubation was carried out overnight (12–16 h) in a humidified chamber using a Petri dish lined with a wetted Kimwipe, with the tissue on the slide placed face-up.

Matrix application was performed using the HTX SubliMATE (HTX Technologies LLC, Chapel Hill, NC) with CHCA (10 mg in 1.5 mL acetone) at an approximate density of 61.33 µg cm^{-2}. Samples were stored in a nitrogen box, and recrystallization was performed prior to MSI analysis as follows: a Kimwipe wetted with 1 mL of 5% isopropanol (IPA) was placed inside a Petri dish, and the slide was attached to the lid with the tissue facing downward. The Petri dish was sealed with Parafilm and incubated at 55–60 °C for 2 min.

Data analysis. Imaging data were imported and extracted from .d Bruker data format via vendor-supplied software SCiLS Lab (version 2025b., Bruker Daltonics, Bremen, Germany). Spectra and peak integration were exported as comma-separated values (.csv). The data were further subjected to visualization and statistical analysis via RStudio (2024.04.2 version), Microsoft Excel, or MetaboAnalyst 6.0.[4] For statistical analysis, ion signals were normalized to the total ion intensity within each sample.

Glycolipid identification criteria. Glycolipids were identified using a multi-parameter approach incorporating the following criteria: (1) selective PLC digestion to remove abundant glycerophospholipids that dominate conventional lipidomic profiles; preliminary validation experiments confirmed no significant loss of target glycolipid signals before and after PLC treatment, and a PLC-processed lipid reference table was generated; (2) high mass accuracy matching to the LIPID MAPS database within ±3 ppm (with noted exceptions explicitly indicated in the identification table); (3) TIMS-based ion mobility separation, in which GlcCer consistently exhibit larger CCS than GalCer at identical m/z values; and (4) biologically consistent spatial distributions across brain regions, as exemplified by the regional localization patterns of HexCer 36:1;O2 (Figure 3).

Supplemental method discussion on laser setting

Instrument parameters were further tuned for glycolipid sensitivity (Table S5). The MALDI-2 laser was operated with a default 5 µs delay. Laser field size settings comparisons showed both 50 µm and 100 µm settings resolved brain morphology specific spatial analyte distributions ; however, the 50 µm setting led to reduced signal because of the smaller ablated area per laser shot and increased acquisition time (Figure 3b). We used the 100 µm setting for replicate imaging to enable statistical analyses and full-section coverage, while reserving the 50 µm setting for select samples to visualize localized differences in isomeric glycolipid metabolism.

In addition, MALDI-2 post-ionization provided only a modest additional sensitivity gain, and this gain was not observed across all species. This may be related to the fact that DAN has a maximum absorbance around 355 nm, whereas the MALDI-2 laser operates at 266 nm.[5, 6] Nevertheless, we observed more consistent signal and modest signal gains for a few species when the MALDI-2 laser was on. It is possible that the MALDI-2 laser functioned partly as a cleanup laser, inducing some fragmentation and/or providing limited additional ionization to the MALDI plume mixture. Because we cannot

definitively identify the mechanism responsible for this behavior, we did not include this discussion in the main text.

SI References

(1) Kleven, H.; Bjerke, I. E.; Clascá, F.; Groenewegen, H. J.; Bjaalie, J. G.; Leergaard, T. B. Waxholm Space atlas of the rat brain: a 3D atlas supporting data analysis and integration. *Nat. Met.* **2023**, *20* (11), 1822-1829. DOI: 10.1038/s41592-023-02034-3.

(2) Nemes, P.; Rubakhin, S. S.; Aerts, J. T.; Sweedler, J. V. Qualitative and quantitative metabolomic investigation of single neurons by capillary electrophoresis electrospray ionization mass spectrometry. *Nat. Protoc.* **2013**, *8* (4), 783-799. DOI: 10.1038/nprot.2013.035.

(3) Asadian, M.; Croslow, S. W.; Trinklein, T. J.; Rubakhin, S. S.; Lam, F.; Sweedler, J. V. High-Throughput Fluorescence-Guided Sequential Single-Cell MALDI-ICC Mass Spectrometry. *Anal. Chem.* **2025**, *97* (29), 15864-15872. DOI: 10.1021/acs.analchem.5c02092.

(4) Pang, Z.; Lu, Y.; Zhou, G.; Hui, F.; Xu, L.; Viau, C.; Spigelman, Aliya F.; MacDonald, Patrick E.; Wishart, David S.; Li, S.; et al. MetaboAnalyst 6.0: towards a unified platform for metabolomics data processing, analysis and interpretation. *Nucleic Acids Res.* **2024**, *52* (W1), W398-W406. DOI: 10.1093/nar/gkae253.

(5) Wang, G. Optical and electrochemical investigation of diaminonaphthalene derivatives. *Synth. Met.* **2010**, *160* (7), 599-603. DOI: https://doi.org/10.1016/j.synthmet.2009.12.013.

(6) Dong, W.; Shen, Q.; Baibado, J. T.; Liang, Y.; Wang, P.; Huang, Y.; Zhang, Z.; Wang, Y.; Cheung, H.-Y. Phospholipid analyses by MALDI-TOF/TOF mass spectrometry using 1,5-diaminonaphthalene as matrix. *Int. J. Mass Spectrom.* **2013**, *343-344*, 15-22. DOI: https://doi.org/10.1016/j.ijms.2013.04.004.
